# NF-κB: At the Borders of Autoimmunity and Inflammation

**DOI:** 10.3389/fimmu.2021.716469

**Published:** 2021-08-09

**Authors:** Laura Barnabei, Emmanuel Laplantine, William Mbongo, Frédéric Rieux-Laucat, Robert Weil

**Affiliations:** ^1^INSERM UMR 1163, Laboratory of Immunogenetics of Pediatric Autoimmune Diseases, Imagine Institute Paris Descartes Sorbonne Paris Cité University, Paris, France; ^2^Sorbonne Universités, Institut National de la Santé et de la Recherche Médicale (INSERM, UMR1135), Centre National de la Recherche Scientifique (CNRS, ERL8255), Centre d’Immunologie et des Maladies Infectieuses CMI, Paris, France

**Keywords:** NF-κB, autoimmunity, autoinflammation, immune tolerance, ubiquitination, genetic diseases

## Abstract

The transcription factor NF-κB regulates multiple aspects of innate and adaptive immune functions and serves as a pivotal mediator of inflammatory response. In the first part of this review, we discuss the NF-κB inducers, signaling pathways, and regulators involved in immune homeostasis as well as detail the importance of post-translational regulation by ubiquitination in NF-κB function. We also indicate the stages of central and peripheral tolerance where NF-κB plays a fundamental role. With respect to central tolerance, we detail how NF-κB regulates medullary thymic epithelial cell (mTEC) development, homeostasis, and function. Moreover, we elaborate on its role in the migration of double-positive (DP) thymocytes from the thymic cortex to the medulla. With respect to peripheral tolerance, we outline how NF-κB contributes to the inactivation and destruction of autoreactive T and B lymphocytes as well as the differentiation of CD4^+^-T cell subsets that are implicated in immune tolerance. In the latter half of the review, we describe the contribution of NF-κB to the pathogenesis of autoimmunity and autoinflammation. The recent discovery of mutations involving components of the pathway has both deepened our understanding of autoimmune disease and informed new therapeutic approaches to treat these illnesses.

## NF-κB Pathway: General Mechanism of Activation

NF-κB is a transcription factor implicated in apoptosis, viral replication, tumorigenesis, inflammation and various autoimmune diseases. The activation of NF-κB is thought to be part of a stress response as it is activated by a variety of stimuli that include Bacterial and viral infections (e.g., through recognition of microbial products by receptors such as the Toll-like receptors), proinflammatory cytokines and antigen receptor engagement, which all lead to activation of NF-κB (1).

The transcription factors NF-κB consist of homo or heterodimers of different subunits that belong to the Rel family of proteins (p65 (RelA), RelB, c-Rel, p105/p50 (NFκB1), and p100/P52 (NFκB2) (2). NFκB1 (p50) and NFκB2 (p52) lack C-terminal transcriptional activation domains (TADs), and their homodimers are thought to act as repressors. As a consequence of their lack of a C-terminal TAD, NF-κB dimers composed of only p50 and/or p52 subunits fail to activate transcription *in vitro* or *in vivo* (3). In contrast, RelA, Rel-B, and c-Rel carry transcriptional activation domains, and with the exception of Rel-B that can only form dimers with p50 and p52, are able to form homo- and heterodimers with the other members of this family of proteins. Excluding RelA, which expression is under the control of a housekeeping promoter, transcription of the genes encoding the NF-κB polypeptides is upregulated by NF-κB, generating a positive feedback response upon cell stimulation (4). In most resting cells, Rel/NF-κB proteins are maintained inactive in the cytoplasm through their association with inhibitory proteins, IκBs. To date, there are eight known IκB (Inhibitors of NF-κB) molecules identified in murine and human cells. These proteins are grouped in three classes (5). p105 and p100, act as inhibitors and precursors of the p50 and p52 subunits. They have the ability to function as Rel protein inhibitors, because they can dimerize with other NF-κB molecules *via* their RHDs domain, whereas their carboxy-terminal Ankyrin repeats serve the function of inhibitory proteins (2). The canonical NF-κB pathway is activated by various signals such as proinflammatory signals (cytokines receptors such as IL-1R and TNF-R family), toll-like receptors (TLR) and T and B cell receptors (1, 6). Upon cellular exposure to these agents, extracellular and intracellular receptors trigger signal transduction events that lead to the activation of the IKK complex through the recruitment of various kinases and enzymes involved in ubiquitin chain formation. These different pathways are detailed below. Once activated, IKKβ phosphorylates and targets IκBs for degradation. IκBs phosphorylation allows the recruitment of the E3 ubiquitin ligase SCF/βTRCP, thus marking them for degradation *via* the 26S proteasome. Then NF-κB is released into the nucleus where NF-κB mediated-transcriptional activation occurs (7). The primary NF-κB effectors of the canonical pathway are transactivation domain-containing polypeptides RelA/p65 and cRel, which form either homodimer or heterodimer with the transactivation domain-lacking p50. Interestingly, many studies have shown that phosphorylation of NF-κB subunits have an impact on their transactivation potential (8). Phosphorylation of RelA by IKKα at Ser536 (Ser534 in mice) is an important mechanism for the negative regulation of pro-inflammatory gene expression (9). The Inhibitors of NF-κB Kinase (IKK) complex consist of the catalytic subunits IKKα, IKKβ, and regulatory subunit NEMO, for NF-κB essential modulator, which is also called IKKγ in human. Biochemical experiments assigned the cytosolic IκB kinase activity to a large protein complex of 700-900 kDa capable of specifically phosphorylating IκBα on Serine 32 and 36. IKKα and IKKβ, 85 and 87 kDa respectively, are ubiquitously expressed. Genetic studies of the knockout mice IKKα^-/-^ and IKKβ^-/-^ showed the physiological significance of these two kinases *in vivo*. Thus, IKKβ-deficient mice were unable to activate NF-κB upon stimulation with proinflammatory cytokines such as TNF-α and IL-1. IKKβ^-/-^ mice exhibit embryonic lethality between days E12, 5 and E14, due to severe liver apoptosis as a consequence of a defective TNF signaling to NF-κB in the liver development (10–12). IKKα-deficient mice died quickly prenatally. These mice exhibit severe defects in multiple morphogenetic events, including deregulation in epidermal differentiation and development, as well as limb and skeletal patterning (13, 14). However, there was no impairment of NF-κB activation induced by either IL-1 or TNF-α in the cells lacking IKKα. These studies also showed an important role of IKKα in the proliferation and differentiation of epidermal keratinocytes (15). NEMO was cloned in Alain Israël’s laboratory in 1998. Then, the human homolog of NEMO was identified biochemically as a component of the IKKα and β containing-complex (16). NEMO knock-out mice studies showed the important role played by this protein in the activation of the IKK complex and NF-κB pathway. Similar to IKKβ^-/-^ mice, the invalidation of *NEMO* gene causes the death of the mice during embryogenesis from liver damage through apoptosis (17). Because NEMO localized on the X chromosome, female mice deficient for NEMO expression survived, because of the X-chromosome dizygosity, but showed a developmental defect of the skin. This abnormality resembles the human disease referred to as Incontinentia Pigmenti (IP), with massive granulocyte infiltration and hyperproliferation and increased apoptosis of keratinocytes (18–20). Another human disorder, anhidrotic ectodermal dysplasia with immunodeficiency (EDA ID), has been associated with mutations of NEMO. This disease is characterized by abnormal development of ectodermal tissues including the skin, hair, teeth, and sweat glands (21, 22).

### Inducers of NF-κB Signaling Pathways Involved in Autoimmunity

The canonical NF-κB pathway is activated by proinflammatory signals (cytokine receptors such as IL-1R and the TNFR family), toll-like receptors (TLRs), and the engagement of lymphocyte receptors.

There is also a non-canonical NF-κB pathway which is mediated by a NEMO- and IKKβ-independent IKKα dimer complex. This pathway is triggered by a subset of TNF family members including CD40 ligand (CD40), B cell Activating Factor (BAFF), and lymphotoxin β (LTβ). This pathway leads to the activation of the protein kinases NIK (NF-κB inducing kinase) and IKKα (23, 24).

#### TCR/BCR-Mediated NF-κB Signaling

**Figure 1:**
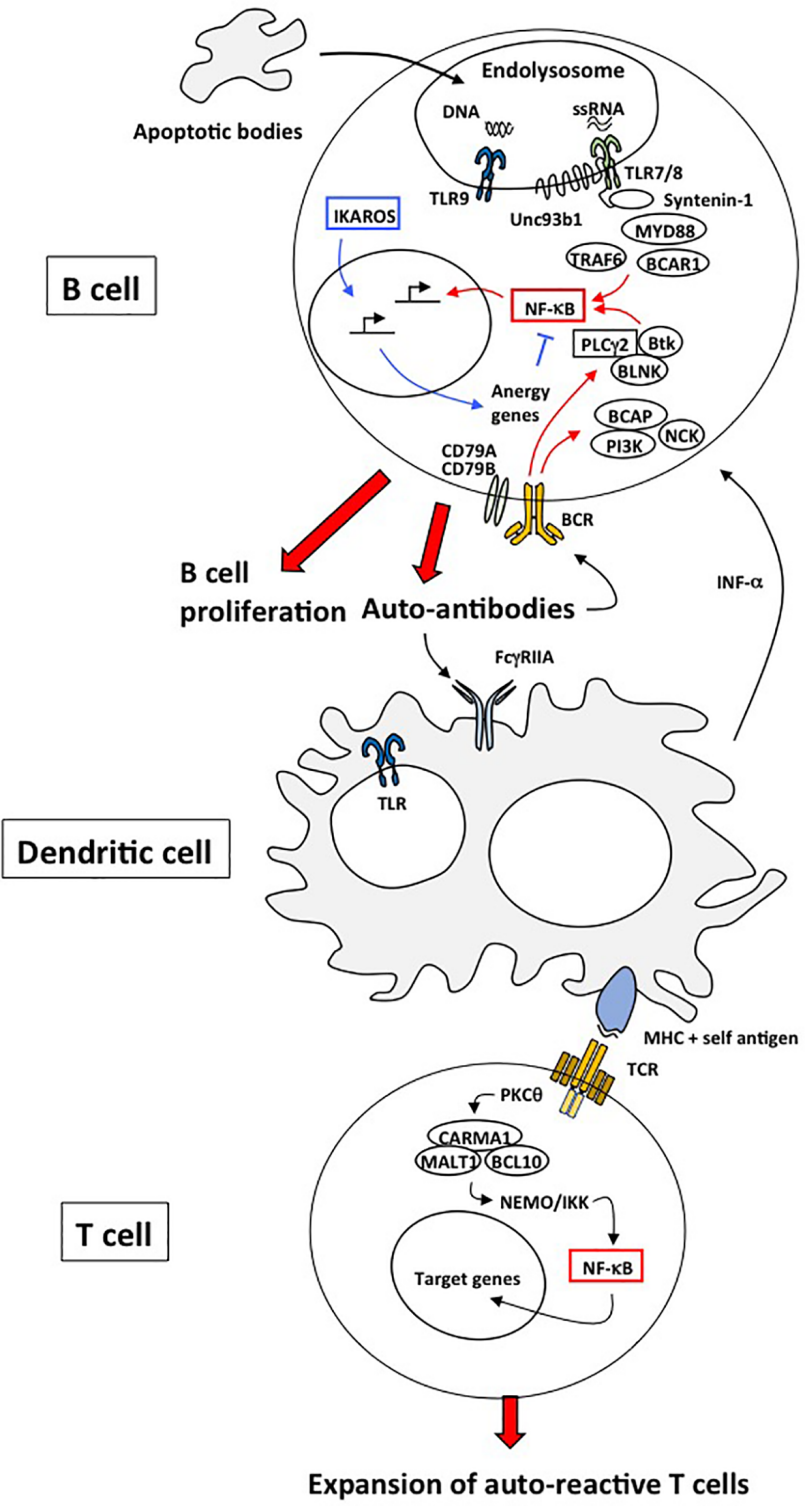
Mechanisms responsible for autoimmunity involving TLRs, BCR and TCR. In B cells, defective clearance of apoptotic cell debris releases nucleic acids capable of activating endosomal TLR7 and TLR9. TLR7- and TLR9-mediated NF-κB activation is dependent on MyD88, IRAK family of kinases and TRAF6. The chaperone UNC93B1 limits TLR7 signaling through its interaction with Syntenin-1 but not TLR9 and thus prevents TLR7-mediated autoimmunity. These TLR receptors can operate in conjunction with the BCR to activate NF-κB in order to increase the production of antibodies. Another transcription gene, Ikaros, induces the expression of anergy genes of which certain inhibit NF-κB. As a consequence, mutations of Ikaros or anergic genes can be responsible of autoimmune diseases. Dendritic cells induce peripheral tolerance by directing the fate of antigen-specific T cells. DCs can present self-antigens to T cells, providing transient T cell activation that can lead to either anergy or deletion of these T cells. DC-mediated tolerance is thus an active process that requires TCR-mediated NF-κB signaling.

In T and B cells, recognition of an antigen by the T Cell Receptor (TCR) or activation of the B cell receptor (BCR) induces the interaction of CARMA1 with a preassembled dimer of MALT1 and BCL10 following its phosphorylation by Protein Kinase C-θ (PKC-θ) or β (PKC−β)s. Thereafter, the tripartite CARMA1-BCL10-MALT1 (CBM) complex activates the NF-κB pathway, leading to the transcription of genes that promote T cell survival and proliferation (**Figure 1**). The activation of NF-κB is highly regulated and requires the post-translational modification of the CBM components through phosphorylation and ubiquitination events. Among the recruited ubiquitin regulators are the E2 ubiquitin-conjugating enzymes UBC13 and UEV1A, the E3 ubiquitin-ligases TRAF2, TRAF6, and cIAP1/2, and the linear ubiquitination assembly complex LUBAC (25). BCL10 undergoes K63-linked ubiquitination through its association with cIAP1 or cIAP2. However, LUBAC and MALT1 undergo K63 ubiquitination mediated by TRAF6. These ubiquitinated proteins recruit the NEMO/IKK complex and ubiquitin-binding proteins TAB2 and TAB3 as well as the kinase TAK1 (25). This culminates in the phosphorylation and activation of IKK kinases, the degradation of IκBα, and the release of the NF-κB (p65/p52). The proteolytic activity of MALT1 is thought to regulate the amplification, duration, and inactivation of NF-κB signaling (25). The end result of this signaling cascade is the production of inflammatory cytokines (TNF, IL-1β, IL-6), chemokines (CXCL8 and CXCL20), and cell survival and proliferation factors (CCND1, BCL2L, and IRF4) (25).

#### TLR-Mediated NF-κB Signaling

Toll-like family receptors (TLRs) are pattern recognition receptors (PRRs) that play a key role in innate and adaptive immune responses. They recognize molecules associated with pathogens as well as endogenous nucleic acids and proteins. They also recognize damage-associated molecular patterns (DAMPs) such as saturated fatty acids and amyloid β. The activation of these receptors leads to the expression of inflammatory genes which play a protective role against infection (26).

TLRs are a family of type 1 trans-membrane glycoproteins with extracellular domains that contain leucin-rich-repeat (LRR) motifs and a toll/interleukin-1 receptor (IL-1-R)-interacting (TIR) domain. There are 11 different TLRs in the human genome; each of these receptors for recognize specific molecular patterns. These patterns include viral and bacterial elements, or endogenous nucleic acids such as ssRNA, dsDNA, and unmethylated cytosine phosphate guanine (CPG)-containing DNA. When TLRs are activated by their respective ligands, they recruit MyD88 to activate NF-κB and IRF transcription factors. This leads to the activation of genes coding for various cytokines and type I interferons. TLR receptors also play an important role in the establishment of adaptive immunity because of the production of pro-inflammatory cytokines. Given their role in innate and adaptive immunity, their sustained activation participates in the pathogenesis of autoimmune diseases.

Another factor in the pathogenesis of autoimmune diseases is defective clearance of apoptotic cell debris (27) (**Figure 1**). The presence of nucleic acids in the cytoplasm due to ineffective clearance activates endosomal TLR7 and TLR9; this is a major cause of systemic lupus erythematosus (SLE). Several mouse models have shown that TLR7 and TLR9 play crucial roles in the production of autoantibodies (28) (**Figure 1**). Their signaling is dependent on MyD88, which binds to IRAK4, activating IRAK1 and IRAK2. IRAK1 and IRAK2 then interact with TRAF6. Upon K63-linked poly-ubiquitination of TRAF6, IRF7 and NF-κB transcription factors are activated.

*Tlr7* transgenic mice spontaneously develop SLE-like phenotype (29). However, production of anti-double-stranded DNA and anti-chromatin autoantibodies that characterize the MLR-*lpr*/*lpr* murine lupus model was diminished by *Tlr9* deficiency, but the mice’s glomerulonephritis was exacerbated (30). Genome-wide association studies (GWAS) have identified different genes and loci associated with SLE. For instance, the B-cell scaffold protein with ankyrin repeats, BANK1, has been linked to SLE (31). Recently, it was shown that BANK1 interacts with two effectors of NF-κB pathway, TRAF6 and MyD88 (**Figure 1**). The D2 isoform of BANK1 is unable to undergo K63-linked ubiquitination; it confers protection against SLE (32). IKZF1, which codes for the transcription factor Ikaros, is also part of the risk genes in agreement with its role in the regulation of many genes involved in anergy (**Figure 1**) (33, 34). Consequently, the invalidation of its expression in mice is responsible for autoimmune diseases (35). TLR signaling is overactive in the B lymphocytes of these mice as a result of a lack of inhibition of MyD88-NF-κB signaling by the Ikaros target genes *Irak3, Tnfaip3* and *Traf1*. Indeed, these 3 genes encodes for negative regulators of NF-κB signaling pathway: IL-1 receptor-associated kinase 3 (IRAK3) inhibits IRAK1/4 at the MyD88 complex; *Tnfaip3* codes for the deubiquitinase A20, which removes poly-ubiquitin chains from TNF receptor-associated factor 6 (TRAF6); TRAF1 interferes with the linear ubiquitination of NEMO by the LUBAC complex.

Several signaling molecules downstream of BCR synergize with TLR pathways to modulate the TLR response. This results in B cell proliferation and the production of autoantibodies. TLR7 and TLR9 can operate in conjunction with BCR (28). Subsequent downstream signals activate NF-κB and MAPK. Many of these signaling effectors potentiate the participation of TLR in autoimmunity (**Figure 1**). Together, these data suggest a crucial role for BCR in regulating TLR responses to maintain normal immune response and prevent pathological B cell activation. Since these receptors both activate NF-κB signaling, it is likely that their synergistic activation acts by potentiating NF-κB activity (36).

#### IL-1R Family-Mediated NF-κB Signaling

The IL-1R family is comprised of 10 receptors. Each of these receptors has multiple variations due to alternative splicing and proteolytic cleavages. The receptors possess 3 Ig-like domains (D1, D2 and D3) as well as intracellular TIR domains (Toll interleukin-1 receptor homology region) that they share with TLR receptors.

Five types of complexes have been distinguished according to the combinations of association between the different chains. Importantly, they all activate NF-κB. Additionally, their deregulations are associated with autoinflammation or autoimmune diseases. Although the involvement of NF-κB in these pathologies is not systematically studied, it is likely a major element.

The IL-1R complex has different functional effects: the induction of proinflammatory cytokines, the production of toxic reactive oxygen and nitrogen species (ROS and RNS), and the generation of prostaglandins (37). The IL-33R complex binds IL-33, which is a Th2 promoting inflammatory cytokine secreted by mast cells, keratinocytes, epithelial cells, and endothelial cells. This receptor complex is overexpressed in patients with severe asthma (38). The IL-36R complex presents a strong association with psoriasis (39, 40). The IL-18R complexes are activated by IL-18 which is implicated in autoimmune diseases such as multiple sclerosis, myasthenia gravis, rheumatoid arthritis, psoriasis, Beçet’s syndrome, autoimmune thyroidis, Crohn’s disease, and type II diabetes (41).

#### NF-κB Activation by IL-1R Receptors

**Figure 2:**
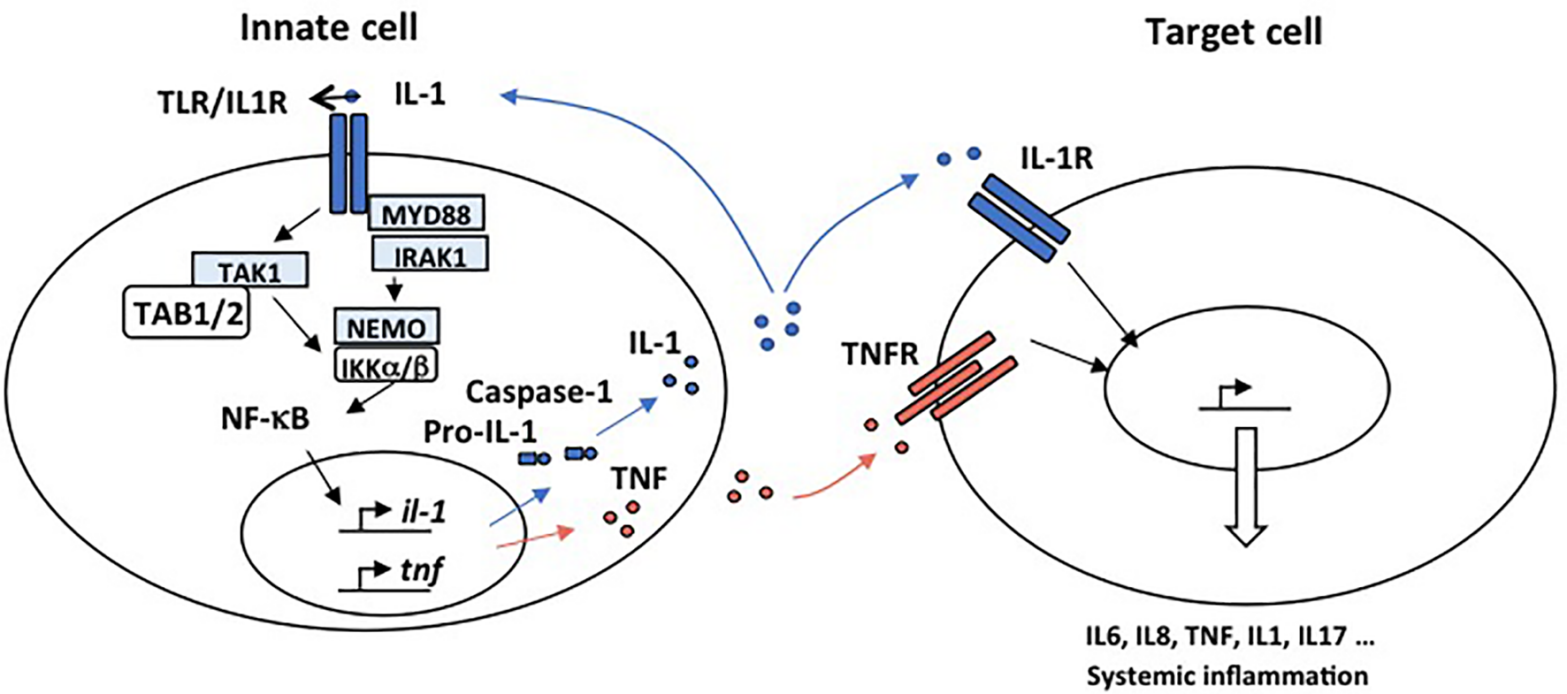
Mechanisms responsible for autoimmunity involving IL-1R and TNFR. IL-1 and TNFα are proinflammatory cytokines able to promote systemic inflammation. Activation of the IL1R complex is part of the innate immune response and plays a major role in setting up the adaptive response. An “inflammatory cascade” leading to the cleavage of IL-1β by Caspase 1 activates this complex. They are important receptors for the initiation of inflammation. All IL-1R contain, like TLR, a TIR domain that intact with MyD88 through an homotypic interaction and drive NF-κB activation. IL-1 is well recognized for its role in the pathogenesis of disorders of autoinflammation. In autoinflammatory diseases, the effector cell is a myeloid cell, characteristically a monocyte or macrophage. IL-1 plays a key role in the expression of several pro-inflammatory cytokines. TNFα is a potent proinflammatory master cytokine modulating inflammatory processes, and its rapid induction is fundamental for the orchestration of the immune response. Monocytes and macrophages are important cellular mediators of TNF-mediated signal transduction. Chronic TNF stimulation leads to inflammatory diseases in which NF-κB is activated such as autoimmunity.

All receptors of the IL-1R family share TIR domains with members of the TLR family such IL-1R and MyD88. TIR domains are required for activation of NF-κB. MyD88 forms an oligomeric complex called the myddosome which transmits receptor-mediated activation of the IL-1R family. To do so, MyD88 recruits the IL-1R-associated kinases, IRAKs (IRAK1, IRAK2, IRAK3 and IRAK4), E3 ligases which comprise TRAF6, Pellino1&2, and the LUBAC complex. Pellino-1&2 and TRAF6 subsequently activate the TGFβ1-activated kinase 1 (TAK1) kinase and facilitate the activation of the NEMO/IKK complex (**Figure 2**).

#### NOD1 and NOD2-Mediated NF-κB Signaling

NOD1 and NOD2 (Nucleotide-binding Oligomerization Domain-containing protein 1 and 2) are important intracellular pattern recognition receptors (PRRs) needed to sense and control intracellular bacteria. NOD1 and NOD2 detect intracellular bacteria through their interactions with peptidoglycans present at their cell surface. While NOD1 recognizes -D-glutamyl-meso-diaminopimelic acid found mostly on gram-negative bacteria, NOD2 detects muramyl dipeptide (MDP) present in the peptidoglycans of most bacteria (42, 43). Once activated, NOD1 and NOD2 trigger immune responses through the activation of NF-κB, mitogen-activated protein kinases (MAPKs), autophagy, and the inflammasome (44). After sensing bacterial elements through their LLR domains, NOD1/2 oligomerize and expose their CARD domains which allow for the recruitment of RIPK2. Multiple genetic alterations of NOD2 have been linked to severe inflammatory conditions including Blau syndrome (BS). BS is an inflammatory disorder that begins in childhood and primarily affects the skin, joints, and the eyes (45). Non-inherited NOD2 gene mutations can also cause early-onset sarcoidosis (EOS), a disease similar to Blau syndrome (46). NOD2 mutations found in BS and EOS are in the NACHT-domain and lead to a spontaneous oligomerization of the protein. As a result, mutated NOD2 leads to NF-κB hyper-activation even in the absence of stimulation by MDP and other bacterial wall stimuli (47). Variations in the NOD2 gene have also been associated with an increased risk of Crohn’s disease, a complex disorder that causes inflammation of the digestive system (48). In contrast to the sporadic mutations of NOD2 found in BS, the mutations of NOD2 involved in CD are all inherited (autosomal recessive). These inherited mutations impair the ability of NOD2 to recognize bacterial particles, thus enabling microbes to proliferate and invade the intestinal mucosa (49). They lead to defective NF-κB activation and altered intestinal bacteria clearance (50, 51).

#### TNFR Family Stimulation and NF-κB Activation

Cytokines of the TNF family play an important role in the coordination of the immune system against pathogens and tumor cells. The 1p36 region encodes TNF receptors with a co-stimulatory role. These include GITR (Glucocorticoid-Induced Tumor necrosis Factor), OX40, HVEM (Herpes Virus Entry Mediator), DR3 (Death Receptor 3), 4-1BB, CD30, and TNFR2 (Tumor Necrosis Factor 2). The role of these receptors as agonists in the immune response makes them prime targets for the treatment of autoimmune diseases. One of the key features of TNFR family receptors is that they all activate NF-κB. Most of them are associated with autoimmune diseases.

**TNFR2** is activated by TNF-α. Its engagement allows activation of NF-κB transcription factor and solicits TRAF2, TRAF3, and TRAF5 E3 ligases whereas **TNFR1** interacts through its death domain with TRADD, a molecule involved in the activation of caspases and apoptotic mechanisms. Interestingly, high levels of TNF-α and TNFR are found in autoimmune diseases. Furthermore, a class III TNF-α polymorphism is associated with a significant number of autoimmune diseases such as Sjögren’s syndrome (52), systemic lupus erythematosus (53), rheumatoid arthritis (54) and ulcerative colitis (55).

**GITR** is a co-stimulatory receptor expressed in antigen presenting cells (APC) and thymic epithelial cells. GITR and GITR ligand expression are correlated with primary Sjögren’s syndrome (56) and thus may have a role in this autoimmune disease.

**DR3** is mainly expressed in activated T cells, APCs, and phagocytes. The expressions of DR3 and its ligand are increased in Crohn’s disease, ulcerative colitis, and rheumatic diseases (57).

**4-1BB** is expressed on activated T lymphocytes as well as endothelial and epithelial cells. It is also involved in autoimmunity such as collagen-induced arthritis, experimental autoimmune uveoretinitis and experimental autoimmune encephalomyelitis (58).

**OX40** is constitutively expressed on ILC types 2 and 3. It is induced following stimulation of naïve T cells. It has a pathogenic role in autoimmune diseases, providing an amplification loop for the generation of autoantibodies in SLE (59).

### Regulation by E3 Ubiquitin Ligases

**Figure 3:**
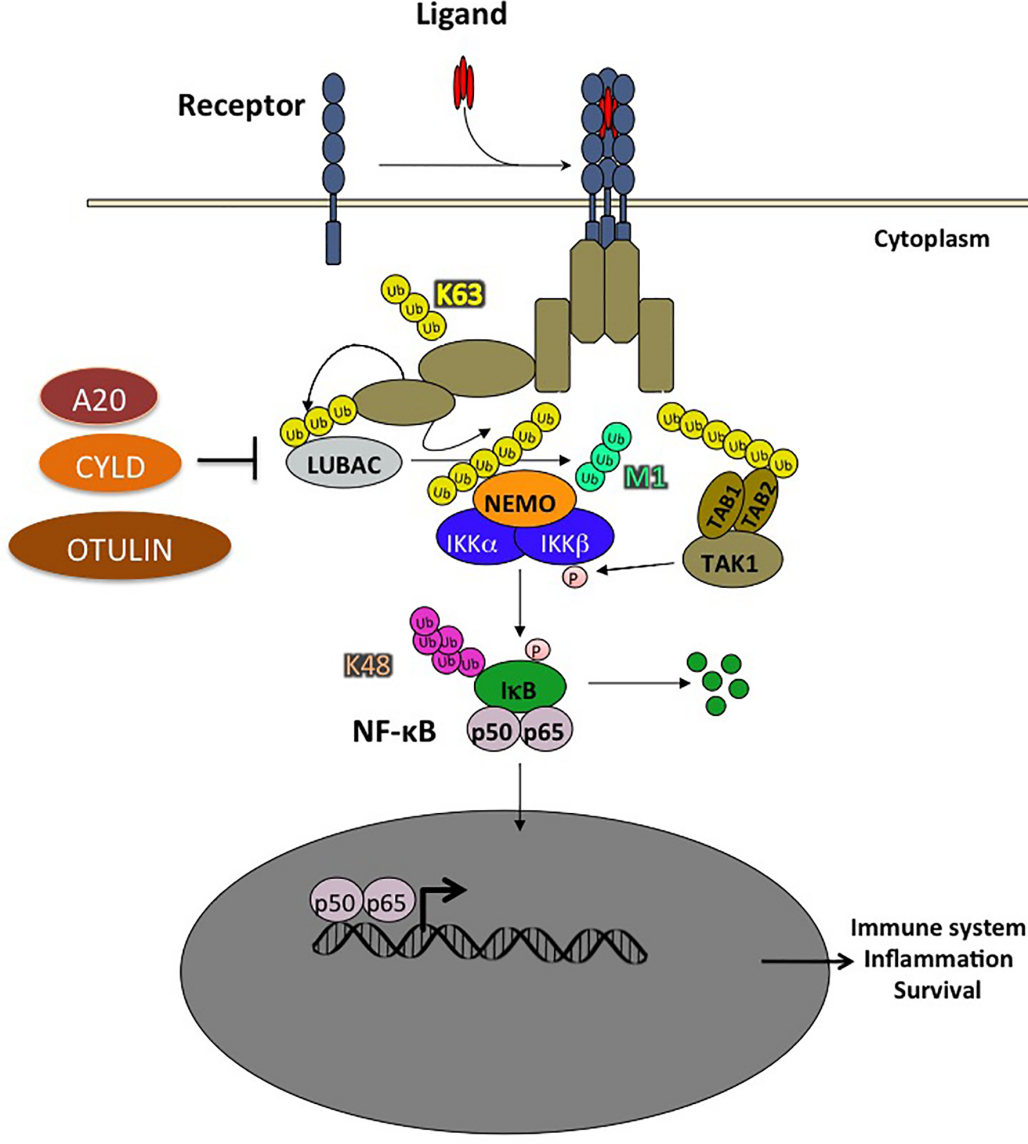
Ubiquitination plays a crucial role in NF-κB activation pathways. The ubiquitination of a substrate can take place with a single ubiquitin (monoubiquitination) or a chain of covalently linked polyubiquitin molecules (polyubiquitination). In the NF-κB pathway, the NF-κB inhibitor, IκB, is modified by K48-linked polyubiquitin chains which are recognized by the 26S proteasome, leading to the degradation of these proteins and the translocation of NF-κB into the nucleus. The polyubiquitin chains linked through the Lysine K63 of ubiquitin do not trigger the degradation of proteins, but rather have non-proteolytic functions such as protein trafficking or activation of kinases and phosphatases. In addition, ubiquitin chains can also be linked linearly, the C-terminal glycine being linked to an N-terminal methionine. Two RING type E3 ligases, HOIL1 and HOIP, specifically assemble linear polyubiquitin chains which play an important role in the regulation of NF-κB. Other types of ubiquitin modifications have also been observed but are not presented in this schema. Ubiquitination is reversible and counter-regulated by a family of deubiquitinases (DUB). There are 3 DUBs playing an important role in the negative regulation of the NF-κB pathway: A20, CYLD and OTULIN, the latter cleaving exclusively linear ubiquitin chains.

NF-κB activation involves many regulatory signaling events among which ubiquitination plays a major role (**Figure 3**). This post-translational modification involves the concerted action of enzymes including E1s (ubiquitin-activating), E2s (ubiquitin-conjugating), and E3s (ubiquitin-ligases). These allow for the covalent attachment of one ubiquitin molecule for mono-ubiquitination, or chains of multiple ubiquitin monomers for poly-ubiquitinaton on a target lysine residue. Ubiquitin units are linked together through one of the seven internal lysine residues of ubiquitin (K6, K11, K27, K29, K33, K48, K63) or, in the case of linear ubiquitination, through the amino-terminal methionine of ubiquitin (also called M1 chains). K48 ubiquitination is a key event in NF-κB signaling that allows the proteasome to degrade IκBs, the inhibitors of NF-κB. More recently, non-degradative ubiquitination has been shown to potentiate NF-κB activation by allowing the recruitment of proteins containing ubiquitin-binding domains (UBDs). The C-terminal coiled-coil domain (CC2) and the LZ domain of NEMO form an ubiquitin binding domain (UBD) that is critical for NEMO function and the activation of the IKK kinases. This domain is called CC2-LZ, UBAN (ubiquitin binding in ABIN and NEMO), or NOA (NEMO Optineurin ABIN) domain. It interacts predominantly with linear ubiquitin chains (60). The C-terminal ZF domain of NEMO is a second UBD which forms a bipartite ubiquitin-binding module together with the CC2-LZ domain, allowing interaction with linear and K63 ubiquitin chains (61).

Regarding M1 chains, a unique ligase complex named LUBAC generates linear poly-ubiquitin chains. LUBAC consists of three subunits (62, 63) namely HOIL-1 (heme-oxidized iron-domains protein (SHANK)-associated RBCK1 homology (RH)-domain interacting protein). Both SHARPIN and HOIL-1 interact with the HOIP UBA domain *via* their ubiquitin-like (UBL) domains (23, 64). LUBAC has been shown to bind to NEMO in the IKK complex. It also conjugates linear chains onto K285 and/or K309 of NEMO. This induces oligomerization of the IκB kinase (IKK) complex leading to activation of IKK by trans-autophosphorylation (65, 66). In addition, LUBAC has other substrates in the NF-κB pathway such as TNFR1, RIP1, RIP2, IRAK1, IRAK2, MyD88, HOIL-1, and SHARPIN themselves following TLR stimulation of macrophages (67, 68).

### Regulation of NF-κB by Deubiquitination

Deubiquitination is a reversible covalent modification catalyzed by deubiquitinases. Three deubiquitinating enzymes (DUBs) were shown to be critical for suppressing NF-κB activation. One of these DUBs is the cylindromatosis tumor suppressor protein, CYLD, which inhibits IKK activation by cleaving K63-linked poly-ubiquitin chains on several proteins, including TRAF2, TRAF6 and NEMO. The C-terminal part of CYLD contains a catalytic USP domain that mediates the cleavage of various poly-ubiquitin linkages. It has a preference for K63 and M1 poly-ubiquitin. Interestingly, CYLD can be inactivated by caspase-8-mediated cleavage at Asp215 in response to TNF and TLR stimulation (69). CYLD has been extensively studied in the context of NF-κB signaling. However, it also plays a role in the JNK and Wnt/β-Catenin pathway. As expected, based on its role in deubiquitinating several NF-κB effectors, CYLD deficiency leads to constitutive NF-κB activation, which results in proinflammatory gene expression. Interestingly, CYLD interacts with the catalytic subunit, HOIP, of the LUBAC complex. It also suppresses linear chain ubiquitination in synergy with OTULIN (70).

Another DUB protein that acts in this pathway is A20 (encoded by the *TNFAIP3* gene). Recent observations have established that A20 mediates its inhibitory function when forming a complex with three other proteins, TAX1BP1, the E3 ligase, Itch, and the E3 ligase, RNF11 (71). A20 belongs to the ovarian tumor (OTU) protease family of deubiquitinating enzymes; these are cysteine proteases that cleave branched or linear ubiquitin chains. A20 is a key negative regulator of NF-κB signaling.

**Figure 4:**
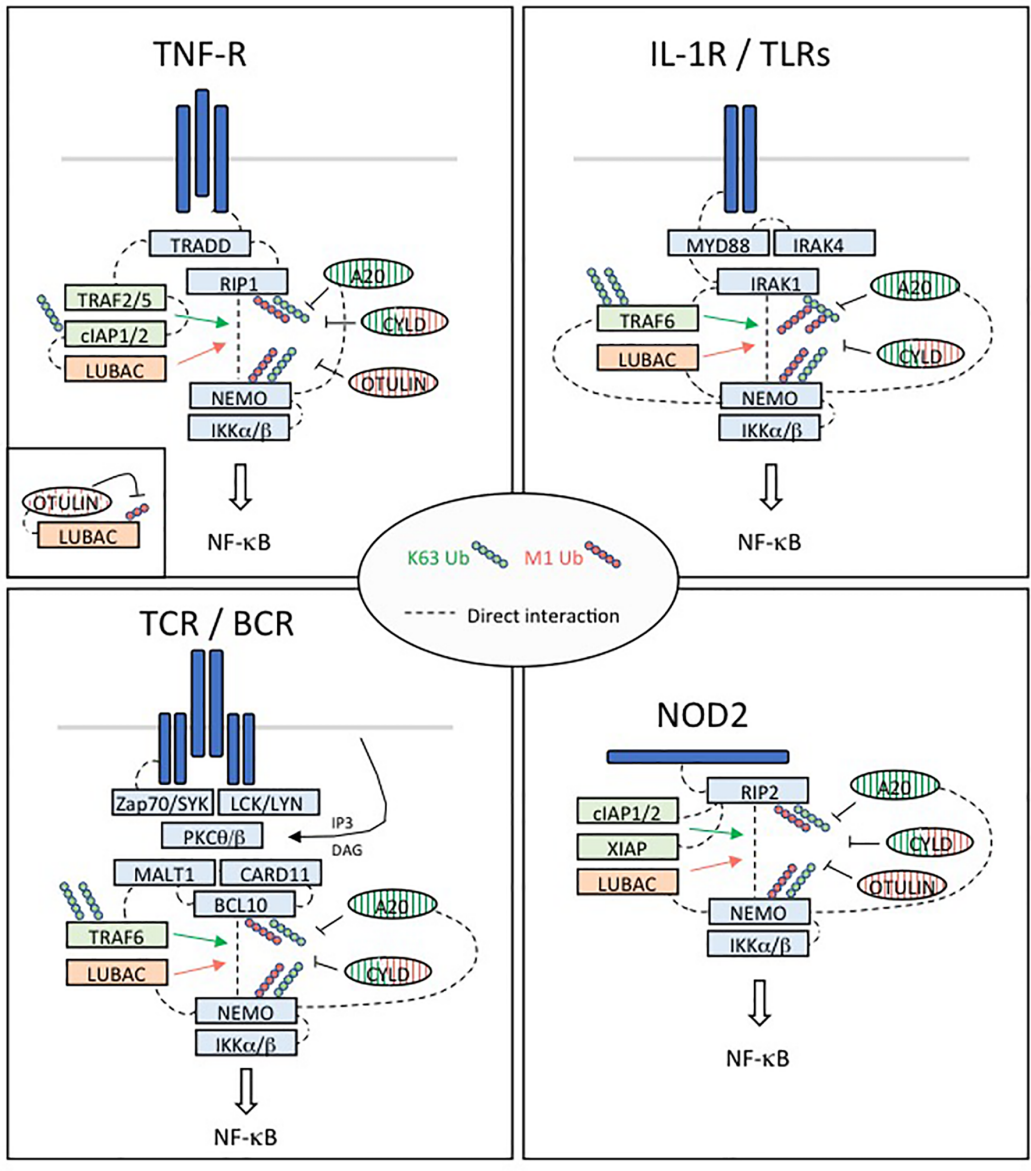
Regulation of NF-κB activation in four different pathways implicated in immune tolerance. **TNFR:** Upon binding of TNFα, the TNFR1 receptor trimerizes resulting in the recruitment of TRADD to the death domain (DD) of the receptors cytoplasmic tail. RIP1 is recruited through TRADD and TNFR1 through homotypic DD interactions. High affinity binding of TRAF2 trimers to TRADD is augmented by TRAF2/RIP1 interactions. TRAF2 trimers recruit cIAP1/2, which in turn recruit the LUBAC complex (HOIP, HOIL-1 and SHARPIN), while RIP1 mediates recruitment of TAK1 and the IKK complex through NEMO. This signaling complex (complex I) signals to NF-κB by recruiting in proximity TAK1 and the IKK complex supporting phosphorylation and activation of IKK. The ubiquitin ligase cIAP1/2 and LUBAC may facilitate TAK1 and IKK activation through production of linear and/or K63 linked ubiquitination. Then, activated IKK phosphorylates IκBα leading its ubiquitination and degradation and nuclear translocation of p65:p50 NF-κB complexes. DNA bound canonical NF-κB induces transcription of immune response genes as well as genes that protect the cell from TNF induced cell death. **IL1R/TLRs:** Despite differences in their extracellular domains, the IL-1 receptor (IL-1R) and TLRs contain a common cytoplasmic motif termed the Toll/IL-1R (TIR) homology domain, which is required for activation of NF-κB signaling pathways. IL-1R- and TLR-mediated NF-κB activation is initiated by the recruitment of MyD88 to the TIR. MyD88 is a scaffold protein that recruits the death domain-containing protein IRAK-1, IRAK-2, IRAK-4, and the ubiquitin protein ligase (E3) TRAF6. IRAK-4 and TRAF6 are essential signaling components of IL-1R- and TLR-mediated MAPK and NF-κB activation. IRAK-1 also plays an important role in IL-1R/TLR signaling in order to induce IKK activation and subsequently IκB activation and NF-κB nuclear translocation. **TCR/BCR:** In response to TCR or BCR triggering, phosphorylation of CARD11 (CARMA1) by PKC-θ or PKC-β on different Serine residues of its linker domain modifies its conformation allowing its association with a constitutively associated dimer formed by BCL10 and MALT1, leading to the assembly of the CBM complex. The formation of this complex constitutes one of the important steps towards NF-κB stimulation following TCR/BCR engagement. In turn, this complex activates the IκB kinase (IKK) responsible for the phosphorylation of the inhibitory factors IκBα and their subsequent degradation, allowing activation and nuclear translocation of NF-κB followed by transcription of its target genes. In addition to its scaffold function during NF-κB signaling, the paracaspase MALT1 exerts a proteolytic activity to ensure the regulation of NF-κB activation. **NOD2:** NOD2 immune function drive a higher incidence of autoimmune diseases such as Crohn’s disease. NOD2-mediated signaling relies on RIP2. RIP2 recruits a number of signaling regulators to the NOD2-associated protein complex, including several ubiquitin E3 ligases such as XIAP, c-IAP1/2, Pellino3, and LUBAC, which promote diverse ubiquitination of RIP2. XIAP is a critical ubiquitin ligase in the NOD2-RIP2 inflammatory pathway and promotes NEMO/IKK- mediated NF-κB activation.

The third DUB, OTULIN, specifically cleaves linear ubiquitin chains. It is the only DUB known to date with such specificity (70, 72). LUBAC is constitutively associated with OTULIN and CYLD, thereby limiting the activity of the LUBAC complex (73) (**Figures 3** and **4**).

## General Introduction on Central and Peripheral Tolerance

Much of the research over the past few decades has centered on the immune system’s involvement in a constant balancing act. To be effective in protecting the host, it must learn to distinguish self from foreign antigen and respond at scale with high specificity to invaders. The immune system is able to recognize self and foreign antigens. Self-reactive T and B cells are present also in healthy people and immune tolerance mechanisms prevent damage to self-tissue and autoimmune diseases. The mechanism of immune tolerance could be central or peripheral. Self-tolerance begins during lymphocyte development and is well established at birth in both mice and humans. The NF-κB pathway has paradoxical roles in the regulation of autoimmunity and inflammation. Particularly, NF-κB is crucial for induction of immune tolerance. It has been shown that, in addition to its transcriptional activity, NF-κB can also activate diverse epigenetic mechanism that mediate extensive chromatin remodeling to target genes to regulate T-cell activities (74).

### The Role of NF-κB in Central Tolerance

Central tolerance concerns immature T or B cells during their differentiation in the primary lymphoid organs, namely the thymus and bone marrow. Impairment of the NF-κB pathway is associated with defects in central tolerance. NF-κB2–deficient mice have impaired T and B cell responses with severe infiltration of lymphocytes into multiple organs and increased activity of autoantibodies to peripheral tissue antigens in a manner similar to that of autoimmune regulator (Aire)-deficient mice (75).

#### Mechanism of Central Tolerance for B Cells

The central tolerance mechanism occurs in the bone marrow when the B-cells are immature and in their early stages of development. Selection can be mediated in various ways at this point of their development:

– **Positive selection:** Initiating maturation, ceasing V (D) J recombination, and migration to the secondary lymphoid organs.
– **Ignoring the expression of its BCR:** B cells continue L-chain rearrangements due to the lack of positive selection signals as a result of low expression or inefficient H-chain–L-chain pairing.
– **Receptor editing or apoptosis:** Can occurs if binds the antigen presented in the bone marrow.

**Figure 5:**
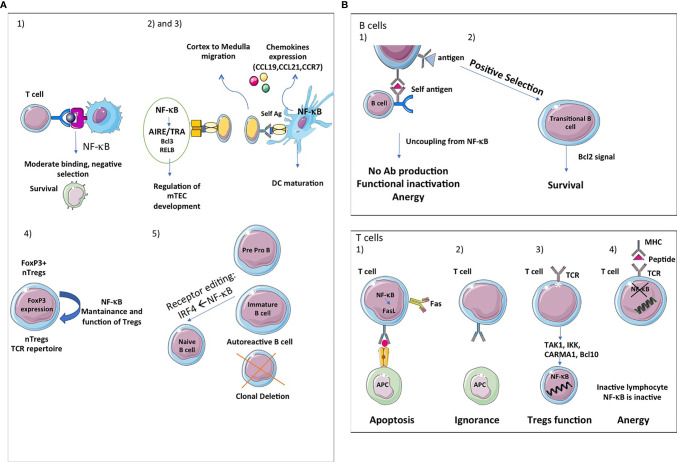
NF-κB, a key player in central and peripheral immune tolerance. **(A) Central tolerance:** The thymic stroma is crucial for the growth, differentiation, positive and negative selection of the T-cell receptor repertoire. The thymic stromal compartment is composed of epithelium, fibroblasts, endothelium, macrophages and dendritic cells, each of which has a distinct role in T-cell development. Single-positive (SP) thymocytes after positive selection have to migrate into medulla to undergo negative selection and further maturation, given the critical role of mTECs in presenting tissue-restricted antigens (TRAs). The thymic medulla is indeed the main site for both negative selection of autoreactive thymocytes and positive selection of Tregs, two important central tolerance mechanisms. (1) First, the classical and alternative pathway of NF-κB activation contributes to the survival of immature cells during the negative selection and thereafter. (2) Second, the alternative NF-κB pathway regulates AIRE/TRAs expression and is involved in mTEC development, homeostasis and function. Bcl3 and RelB also regulate mTEC development at the DNA level. The deubiquitinase CylD is implicated in mTEC differentiation (3). Third, NF-κB also regulates the expression of chemokines (CCL19, CCL21, CCR7) implicated in cortex to medulla migration of DP thymocytes and to secondary lymphocytes organs and tissues. (4) Fourth, the NF-kB pathway has also been shown to influence thymic or natural Treg (nTreg) cells development and function. These cells recognize self-antigens and play an important role in the maintenance of immunological self-tolerance. Forkhead Box P3 (FOXP3) is the main transcription factor for the differentiation of these cells. NF-κB is important for the maintenance and functions of nTregs and the expression of FOXP3. (5) Fifth, in B cells, NF-κB participates in clonal deletion of autoreactive B lymphocytes and receptor editing, a protective mechanism of ongoing gene rearrangement that generates a new receptor with an innocuous specificity that prevents cell death by apoptosis. If the BCR recognizes sel-antigen, it is down regulated under the control of *IRF4*, a target gene of NF-κB. **(B) Peripheral tolerance:** Peripheral tolerance takes place in the periphery and its purpose is to ensure that T cells and B cells that escape from the thymus cannot give rise to autoimmunity. NF-κB signaling is essential for the development and maintenance of secondary and tertiary lymphoids organs. In B cells, peripheral tolerance uses two different mechanisms: (1) Anergy, which is characterized by desensitization of BCR signalling and its uncoupling from the NF-κB pathway, resulting in a failure of antigen presentation and antibody production. (2) NF-κB is essential for the survival of developing B cells in the spleen. Bcl2 is involved in the survival of transitional B cells. The classical and alternative pathways pathway of NF-κB activation are involved in B cells maturation. In T cells, peripheral tolerance uses four different mechanisms: (1) Inactivation of effector T cells by clonal deletion. Programmed cell death (apoptosis) is an essential mechanism leading to clonal deletion of T cells with high affinity for self-antigens. NF-κB plays a pro-apoptotic role in negative selection. (2) T-cells ignore certain self-antigens because they are located in immune-privileged sites or because they have low immunogenicity (low levels of expression or low binding affinity). (3) Conversion of T cells in Tregs. Non canonical NF-κB activation is important for the maintenance of these cells *in vivo via* its effect on mTEC development and canonical NF-κB plays is involved in Foxp3 expression which is fundamental for the function of Tregs. Components of the TCR-mediated NF-κB pathway such as TAK1, IKK, CARMA1 and Bcl10 are also involved in Tregs development. Ubiquitination is fundamental for Tregs functions. (4) Induction of anergy. Anergy is a tolerance mechanism in which lymphocytes are intrinsically functionally inactive. NF-κB is inactive in these unresponsive lymphocytes. NF-κB also controls DCs maturation.

Although the central tolerance mechanism is very efficient, some of the self-reactive clones of B-cells in the bone marrow escape the process and migrate to the periphery (76). During the negative editing/selection phase, the survival of the immature B-cells is contributed by NF-κB (**Figure 5**). The partial reduction of immature B-cells has been documented in mice that lack both NFκB1 and NFκB2 (77). BAFF receptor signaling activates the non-canonical NF-κB pathway (77). The involvement of BAFF receptor signaling in the *de novo* generation of “more mature” immature B-cells in the bone marrow has been implicated by another study as well (78). A reduction in the number of immature B-cells has been observed by the conditional ablation of IKKα/IKKβ or NEMO (79).

#### Mechanism of Central Tolerance for T Cells

Central tolerance in T cells involves several mechanisms, including clonal deletion (death), induction of a refractory state (anergy), inhibition of function by other cells or their products (immune regulation) and sequestration of antigen (ignorance).

The central tolerance takes place in the thymus where T lymphocytes arise from circulating bone-marrow-derived progenitors. T-cell receptor (TCR) gene rearrangement forms either γδ or αβ progenitors at the CD4 and CD8 double-negative (DN) stage. Some αβ DC cells go on to give rise to a pool of CD4 and CD8 double positive (DP) T cells whose antigen specificity is fine-tuned by somatic recombination of TCR genes (80). DP thymocytes expressing TCRs without affinity to self-peptide-MHC complexes perish by neglect. During positive selection, thymocytes with some affinity to self-peptide-MHC complexes form either CD4 or CD8 single-positives (SP) (81).

Positively selected CD4^-^ or CD8^-^ T cells express the chemokine receptor CCR7 and migrate into the medulla, where the CCR7 ligands CCL19 and CCL21 are highly expressed by medullary thymic epithelial cells (mTECs) (82).

In the medulla, CD4^+^ or CD8^+^ T cells interact with antigen-presenting cells, such as mTECs and dendritic cells and these interactions normally result in the deletion of autoreactive T cells. mTECs and thymic dendritic cells delete almost all T cells recognizing self-antigen–MHC complexes with high affinity. This process is called negative selection and it is mainly regulated by mTEC, which provides a microenvironment to test the reactivity of the generated T cells before leaving the thymus. Mutant mice lacking the non-canonical NF-κB members (RelB and NFκB2) or upstream signaling molecules, such as NIK, IKKα and LTβR, have impaired mTEC formation, coupled with autoimmune symptoms (**Figure 5A**) (83). Bcl3 appears to be redundant with NFκB2 for mTEC development (84). In parallel with NF-κB2, Bcl3 functions within stroma to generate medullary thymic epithelial cells, which are essential for negative selection of autoreactive T cells (85).

RelB is influenced by the activity of the canonical NF-κB signaling pathway, which enables its effect on the development, and population of mTEC (86). It has also been shown that the later stages of mTEC differentiation can by altered by CYLD dysfunction, which suggests a role for RANK signaling (87), as shown in **Figure 5A**.

### The Role of NF-κB in Peripheral Tolerance

Not all self-reactive lymphocytes can be eliminated by the central tolerance mechanisms despite the efficiency of these processes. The lymphocytes that escape elimination by the central tolerance mechanisms are fed to other mechanisms in the primary lymphoid organs which are called peripheral tolerance mechanisms.

#### Mechanism of Peripheral Tolerance for B-Cells

Roughly one in ten immature B cells leave the bone marrow to enter the spleen and encounter self-antigens absent in the former compartment (88). While high avidity interactions with these self-antigens result in the deletion of B cells, low or very low avidity interactions lead to anergy and ignorance (78). After exposure to self or foreign antigen, these cells extravasate to the periarteriolar lymphoid sheath (PALS) of the T cell zone (89). Without T cell help or TLR-dependent co-stimulatory signals, B cells, regardless of their specificity perish within three days. Anergy is marked by the desensitization of BCR signaling and dissociation from NF-κB signaling, resulting in decreased access to B cell survival factor, BAFF (**Figure 5B**). BAFF-deficient mice have lower circulating peripheral B cells (90). In contrast, transgenic mice overexpressing BAFF have been shown to develop SLE and Sjogren’s-like illnesses marked by the production of autoreactive antibodies like rheumatoid factor, anti-dsDNA, and other ANAs (91). Some autoreactive new transitional/emigrant B-cells fail to be removed from the B-cell population because of the inhibition of the counter-selection by increased levels of BAFF concentration. In the spleen, the essential component for the development of B-cells is NF-κB (92).

#### Mechanism of Peripheral Tolerance for T Cells

T-cells with mild affinity or low affinity towards self-antigens may escape into the periphery from the thymus despite negative selection. Peripheral mechanisms are required for two main reasons. The first is to check and control lymphoproliferation which is almost always followed by an immune response. The second is to control mature self-reactive T-cells (93). These mechanisms include Fas-mediated apoptosis, causing clonal deletion of T-cells as well as the suppressive function of regulatory T-cells (Tregs) (**Figure 5B**).

#### The Role of NF-κB in the Differentiation of Th1 and Th2

After stimulation, naïve CD4^+^ T cells differentiate into Th1 and Th2 effector cells to facilitate a nuanced immune response that can adapt to a wide array of challenges. Th1 cells are adept at guiding defense against intracellular pathogens through the production of IFN-γ (94). Th2 cells protect against invasion by extracellular microbes and are implicated in allergic reactions (95). They are characterized by their production of an array of cytokines, including IL-4, IL-5, IL-6, IL-9, IL-13, and IL-25. Mice expressing a non-degradable form of IκBα in T cells have been shown to have a reduce Th1 responses resulting from decreased NF-κB activation (1). IFN-γ production is dependent on adequate levels of RelA while RelB plays an important role in Th1 differentiation through T-bet (96). Mice deficient in c-Rel have defective Th1-mediated immune responses and impaired IFN-γ production (97). The development of Th2 cells is initiated by IL-4 stimulation which triggers the activation of the transcription factor STAT-6 (98). NF-κB has been shown to attach to two enhancer sites in the IL-4 locus in contact with nuclear factor activated T cells (NFAT) to stimulate its induction (99). Additionally, NF-κB is involved in the expression of GATA3, a master regulator of Th2 differentiation (100).

#### The Role of NF-κB in the Differentiation of Th17

Th17 cells protect the host against fungal infections and certain extracellular pathogens by playing an important role in the defense system of the host (101). The deficiency of RelA causes the dendritic cells to decrease their production of interleukin-1α, interleukin-1β, and interleukin-6 in response to LPS stimulation and these defects alters Th17 differentiation (102, 103). Moreover, STAT3 promoter activation is mediated by PKCθ which is stimulated by IKKβ (104). This step is essential for the differentiation of Th17. MALT1, one of the components of the antigen-mediated NF-κB activation pathway, also controls the differentiation of T-cells into Th17 cells (102). In addition, the development of Th17 cells is blocked by the deficiency of CARMA1 (103). Furthermore, the differentiation of Th17 cells is impaired by the stabilization of IκBα (This leads to the inhibition of NF-κB activation) when calpastatin (a natural inhibitor of calpain) minimal domain is overexpressed (105).

#### The Role of NF-κB in Regulatory T Cells

Treg function is maintained by NF-κB. NF-κB controls T-cells’ intrinsic actions, mTEC development, and thymic dendritic cell development. The thymic development of Tregs as well as the expression of FOXP3 depend on the c-Rel subunit of NF-κB (106, 107). It has been demonstrated that RelB regulates the development of mature mTEC. Mice with conditional deletion of RelB lacked mature mTECs and developed spontaneous autoimmunity. In addition, the NF-κB subunits RelA and c-Rel, which are both activated by classical NF-κB signaling, were jointly required for mTEC differentiation by directly regulating the transcription of RelB (86). Moreover, mice lacking canonical NF-κB pathway signaling components produce fewer Treg cells. These components include the lymphocyte-specific TAK1/IKK-activating factors CARMA1, IKK, BCL10, and TAK1 (**Figure 5B**). A significant decrease in the development of Treg has been observed in mice that have a deletion in the IKK negative regulator CYLD or express constitutive IKKβ activity (108). The development of Treg can also be impaired by several other factors (83). These include ablation of non-canonical NF-κB signaling. For example, a reduced number of Treg cells are observed in the alymphoplasia (aly) mice which present a mutation in the NIK gene. Using a model of induced Tregs (expression of FOXP3-encoding vector in primary CD4^+^ T cells), it was shown that p65 phosphorylation level and translocation of various NF-κB subunits (p65, p50 and c-Rel) were decreased (109). However, blockade of NF-κB signaling using pharmacological compounds or IκBα expression did not affect the *in vitro* suppressive capacity of Tregs (109). The *in vivo* function of Tregs is compromised by the ablation of the conjugating enzyme that is specific for the K63 ubiquitin chain. This leads to the development of autoimmunity as well as impaired T-cell homeostasis (110). Ubc13 is necessary for MAPKs and the activation of IKK that P12 is TCR-stimulated in conventional T-cells (111). Ubc13 is dependent on the IKK pathway for its Treg-specific functions. In the Ubc13-defective Treg cells, a constitutively active IKKβ encoded by a transgene can largely rescue the functional defect (112). Moreover, TEC-specific ablation of NIK or IKKα blocked medullary TEC development, resulting in production of pathogenic autoreactive T cells due to breakdown of T cell central tolerance (113).

#### The Role of NF-κB in Dendritic Cells

lDCs fall into one of two main groups based on their migratory capacity and location. CD8a^+^, CD11b^+^ conventional DCs (cDCs), and plasmacytoid DCs (pDCs) are included in the resident lymphoid-tissue (LT)-DCs. On the other hand, CD11b^+^ cDC and CD8a-type subsets are included in the non-lymphoid tissues (NLT)-DCs that are found in the parenchyma tissue. It has also been postulated that NLT-DCs undergo a maturation process that leads to the upregulation of CCR7 and their subsequent migration (114). The signaling pathways that regulate NLT-DC maturation have yet to be elucidated. Mice with deletion of IKKβ in DCs lineage have dysregulated immune function and spontaneous autoimmunity. Furthermore, NF-κB signaling is needed for the accumulation of DCs in draining LNs and promotion of immune tolerance to host tissue. Steady-state accumulation of DCs in draining lymph nodes also requires NF-κB signaling (115).

### Immune Checkpoints and Tolerance

An essential regulatory process that controls autoreactive T-cells is mediated by the interaction of coreceptors with their ligands such as the interaction of CTLA‐4 (cytotoxic T lymphocyte antigen 4, CD152) and CD28 with B7‐1 (CD80) and B7‐2 (CD86) and interaction of PD-1 (programmed cell death protein 1) with PD-L1 and PD-L2. An activating stimulus delivered by CD28/B7 signal leads to anti-apoptotic factor expression, proliferation, and interleukin-2 production in response to NF-κB activation. CTLA4A plays a key role in regulatory T cells and central tolerance and its deficiency is sufficient to cause lymphoproliferation and autoimmune diseases (116, 117). PD-1 expression is regulated by NF-κB (mainly in macrophages) and presents a key role in regulating T-cell tolerance and autoimmunity (118–120) Consequently, impaired PD-1-PD-L1 functions have been involved in large variety of autoimmune diseases (119). Interestingly, administration of an immunotoxin consisting of an anti-PD-1 single chain variable fragment coupled to a *Pseudomonas* exotoxin selectively kills PD-1 expressing cells and delay diabetes onset in a mouse model of autoimmune diabetes (120). However, PD-1 immunotherapy is better known for inducing or precipitating autoimmune diseases (121, 122).

## Genetic Defects in the NF-κB Pathway Responsible for Autoimmunity

Primary immunodeficiencies (PIDs) are monogenic diseases offering unique natural models to better understand the role of a given gene and its product. The study of patients presenting with PIDs has revealed a wide variety of genes responsible for autoimmunity and autoinflammation linked to the NF-κB signaling pathway (**Table 1**).

**Table 1:** List of genes mutated in humans linking NF-κB to autoimmunity.

Gene (protein)	Mutations		Inheritance	Mechanism of disease	Phenotype	Refs
*CARD11* (CARMA1)	L194P (het) R975W (het) E57D (het) Dup183_196 (het)		AD, LOF	n.d	Immunodeficiency with severe atopic dermatitis	(123)
*CARD14* (CARMA2)	Many heterozygote variants, rare homozygote cases		AD, GOF	Constitutive NF-κB activation in keratinocytes	Severe forms of psoriasis	(124)
*FAM105B* (OTULIN)	L272P (hom) G174Dfs*2 (hom) G281R (hom)		AR, LOF	Failure to hydrolyze linear ubiquitin chains made by LUBAC. Excessive NF-κB signaling in myeloid cells. Increased apoptosis by TNF signals.	ORAS (OTULIN-related autoinflammatory syndrome/ Otulinpeia). Multi-organ inflammation, autoimmunity	(125, 126)
*IKBKG* (NEMO)	E391X (het)		XLR, GOF	Increased NF-κB activation in response to TNF and Toll-like receptor stimulation due to impaired interaction of NEMO with A20	X-linked ectodermal dysplasia with anhidrosis and immunodeficiency (EDA-ID). Inflammatory skin and intestinal disease	(127)
*IL-1RN* (IL-1-RA)	Z77X (het) Q54X (het) N52KfsX25 (het) Includes also various heterozygotes deletions and truncations.		AD, LOF	Failure to antagonize IL-1R signaling	systemic inflammation with skin and bone involvement.	(128)
*IL36RN* (IL36-RA)	L27P (hom) S113L (hom)		AR, LOF	Failure to antagonize IL-36R signaling	Generalized pustular psoriasis	(128, 129)
*LAT*	G89fs*11 (hom)		AR, LOF	Reduced numbers of T cells. Normal Ca2+ influx and NF-κB signaling in residual T cells but abrogated ERK signaling.	Early onset combined immunodeficiency and autoimmunity	(130)
*LYN*		Y508X (het)	AD, GOF	Constitutive phosphorylation of Lyn in B-cells	Early onset autoinflammatory phenotype. Hepatosplenomegaly, vasculitis, circulating auto-antibodies	(131)
*NFKB1* (P105)		H67R (het) I553M (het) R157X (het)	AD, LOF	NF-κB signaling defect	Variable symptoms of immunodeficiency, autoinflammation and autoimmunity. Similarities with Behçet’s disease.	(132)
*NFKBIA* (IκBα)		S36Y (het) Q9X (het) W11X (het) E14X (het)	AD, LOF	NF-κB signaling defect	Immunodeficiency and non-infectious systemic inflammation (bowel, cutaneous)	(133)
*NOD2*		Many variants in general homozygotes or compound het.	AR, LOF	Compromised NOD2 signaling	Inflammatory bowel disease (IBD)	(134)
*NOD2*		Many heterozygote variants mostly positioned in the central nucleotide-binding and oligomerization domain	AD, GOF	Constitutive activation of NOD2 signaling	Blau syndrome (BS) and early-onset sarcoidosis (EOS). Non-caseating granulomatous inflammatory disease.	(48)
*RBCK1* (HOIL-1)		L41fsX7 (hom) Q185X/del	AR, LOF	Lack of linear ubiquitination. NF-κB signaling defect. Increased apoptosis by TNF signals.	Immunodeficiency, autoinflammation multi-organs, amylopectinosis	(135)
*RIPK1* (RIP1)		I615T (hom) Y426X (hom) M318fs T645M C601Y	AR, LOF	Reduced NF-κB activity, defective differentiation of T and B cells, increased inflammasome activity, and impaired response to TNFR1-mediated cell death in intestinal epithelial cells.	combined immunodeficiency and inflammatory bowel diseases	(136)
*RNF31* (HOIP)		L72P (hom)	AR, LOF	Lack of linear ubiquitination. NF-κB signaling defect. Increased apoptosis by TNF signals.	Immunodeficiency, autoinflammation multi-organs, amylopectinosis, lymphangiectasia	(137)
TNFAIP3 (A20)		L227X (het) F224S fs*4 (het) R271X (het) T604R fs*93`(het) Y306X (het)	AD, LOF	Defect in hydrolyzing K63 ubiquitin chains. Excessive NF-κB P14 signaling	HA20 syndrome (Haploinsufficiency of A20). Similarity with Behçet’s disease. Early onset auto-inflammatory syndrome	(138)
*TNFRSF11A* (RANK)		M416Cfs*110 (het)	AD, GOF	Increase RANK signaling. May increase TNFR1 signaling through RANK crosstalk	TRAP-related syndrome	(139)
*TNFRSF1A* (TNFR1)		C30R (het) C33Y (het) T50M (het) C52F (het) C88R (het) C88Y (het) S59P (het)	AD, GOF	Constitutive TNFR1 activation	TNF Receptor-Associated Periodic Syndrome (TRAPS). Periodic fevers and severe localized inflammation.	(140, 141)
*TRIM22*		R442C (hom) R150T/S244L (comp het) R321K (hom)	AR, LOF	Defective NOD2 signaling due to a lack of K63-ubiquitination of NOD2	Early onset inflammatory bowel disease	(142)
*XIAP*		C203Y (het) P225Sfs*2 (het)	XLR, LOF	Impaired NOD2 signaling. enhanced lymphocyte apoptosis.	Lymphoproliferative disorder XLP2). Inflammatory bowel disease	(143)

### REL Homology Domain Protein Family

Mutations in three of the five RHD protein genes: p50 (*NFKB1*), p65 (*RELA*), and c-Rel (*REL*), have been chiefly associated with humoral immunodeficiency (144). Nevertheless, clinical manifestations can be broader, including autoimmune and/or inflammatory manifestations.

#### NFKB1 Haploinsufficiency: Inflammation/AI CVID

NFKB1/p50 mutations can be classified as haploinsufficiency mutations, precursor skipping mutations with truncation in the central part of p105, missense mutations in the N-terminal part which affect p105 and p50, and missense mutations in the C-terminal part which affect only p105. These mutations can lead to different phenotypes. Loss-of-function of *NFKB1* are the most common monogenic cause of common variable immunodeficiency in Europe associated with lymphadenopathy, splenomegaly and with age, autoimmunity and cancer (145). Other clinical presentation linked to *NFKB1* mutants include respiratory infections, hypogammaglobulinemia with low IgG, IgM and IgA (reflecting deficient Ig isotype class-switching), Epstein-Barr virus (EBV)-associated lymphoproliferation and low peripheral B cell counts (146).

A recent study of Finnish families with loss of function mutations in *NFKB1* identified miscodings affecting protein stability, defective subunit phosphorylation, and translocation of the nucleus causing a wide range of immune disorders (132). Mice without p50 subunit of NF-κB exhibit defects in B cell responses to infection but show no developmental abnormalities (147).

#### NFKB2 and Autoimmunity

Autosomal Dominant (AD) *NFKB2*/p100/p52 mutations have been identified in patients with common variable immunodeficiency (CVID). These patients exhibit recurrent respiratory infections, hypogammaglobulinemia, alopecia, and adrenal insufficiency. They present poor antibody response, unswitched naive B cells, and defective T and NK cell activation (148). Autoantibodies are frequently present in these patients. Mutations in *NFKB2*/p100/p52 impact the processing of p100 to p52 after CD40-CD40L stimuli (149, 150). In this way, p100 cannot form a dimer with p65 in the canonical pathway (151). Because of this, *NFKB2* mutations cause similar effects to *NFKB1* deficiency but with a more severe CID phenotype (152). In addition, *NFKB2* knockout mouse models as well as *NFKB2* mutant, c.2854A**>**T (*NFKB2Lym1/Lym1*, nonsense mutation p.Tyr868*), demonstrate a CVID-like phenotype with hypogammaglobulinemia and poor humoral response to antigens (153).

#### *RELA* Haploinsufficiency

*RELA* haploinsufficiencies have been recently described in patients presenting with oral aphthosis and inflammation or with autoimmune lymphoproliferative syndrome (154). The patient’s’ fibroblasts exhibit increased apoptosis in response to TNF due to defective expression of NF-κB–dependent anti-apoptotic genes (155). *RELA^+/−^* mice have similarly impaired NF-κB activation, develop cutaneous ulceration from TNF exposure, and exhibit severe dextran sodium sulfate (DSS)-induced colitis, ameliorated by TNF inhibition (155).

Another *de novo* heterozygous mutation has been found in a 5-year-old patient with autoimmune lymphoproliferative syndrome (ALPS) (154).

#### RELB

Autosomal recessive *RELB*-deficiency has been found in patients with CID from infancy, showing recurrent infections and severe autoimmune skin diseases with decreased c-Rel nuclear translocation after CD40 ligand stimuli. These patients exhibit an accumulation of memory T cells (156). The defects in T and B cells in patients were not observed in knockout mice which present inflammatory and hematopoietic abnormalities (45, 48).

#### c-Rel

C-Rel is mainly expressed by hematopoietic cells. It is involved in the proliferation and differentiation of lymphocytes. It also acts in the regulation of genes coding for proteins involved in the immune response including IL-2 and FOXP3. Mutation of its inhibitory domain induces an enhancement of c-Rel binding to DNA, therefore increasing its transactivating activity (157).

### Genetic Defects of Regulators

#### CARD

##### CARD11

Genetic defects of *CARD11*, *BCL10*, and *MALT1* have been identified in various primary immune deficiencies with autoimmune and inflammatory features. This illustrates the key role of the CBM complex in immune homeostasis.

Autosomal dominant gain-of-function mutations of *CARD11* lead to massive B cell expansion with NF-κB hyperactivation and T-cells anergy (BENTA syndrome) (158–160), whereas B lymphocytosis and hypergammaglobulinemia are a noted consequence of a constitutive NF-κB activation in B-cells. These patients exhibit hypo-IgM and associated upper respiratory tract infections.

Autosomal dominant loss-of-function *CARD11* mutations have been found in eight patients from four unrelated pedigrees with atopic dermatitis and recurrent infections (123). One patient also presented with ulcerative colitis and transient hypogammaglobulinemia. His T cells exhibited defective activation and proliferation. The signaling defect could be partially remedied by glutamate supplementation. Lastly, autosomal recessive loss-of-function *CARD11* mutations have been found in patients with CID. Whereas the numbers of T- and B-cells were normal, there was a block in the B-cell maturation at the transitional stage as well as defective T-cell functions (161).

##### CARD14

GOF mutations in *CARD14* lead to generalized pustular psoriasis. The discovery of heterozygous *CARD14* mutations in psoriatic diseases revealed an unexpected function of CARD14 in keratinocytes and innate immunity (162). However, CARD14 increased expression and NF-κB hyperactivation have also been observed in psoriasis without *CARD14* mutations (163)

#### Caspase 8

Caspase 8 is a proapoptotic protease with an essential role in lymphocyte activation and protective autoimmunity. *CASPASE 8* deficiency in humans and mice abolishes the activation of the NF-κB after stimuli with Fc receptors or Toll-like receptor 4 in T cells, B cells, and natural killer cells (164). Initially, autosomal recessive *CASPASE 8* mutations had been identified in patients presenting with features of autoimmune lymphoproliferative syndrome (ALPS) such as lymph node and spleen enlargement, FAS-mediated apoptosis defect, mild elevation of CD4^-^ CD8^-^ double negative T cells, and bacterial or/and viral infections. These features were not observed in ALPS-FAS patients (165). Strikingly, *CASPASE 8*-deficiency was also found to cause very early-onset inflammatory bowel disease (VEO-IBD). This condition is associated with lymphocyte dysfunction, impaired inflammasome activation, and defective intestinal epithelial cell death responses (166).

### Genetic Defects of Ubiquitin Ligases and Deubiquitinases

#### Ubiquitin Ligases

##### LUBAC

LUBAC deficiencies have been identified in patients harboring bi-allelic recessive mutations in *HOIL-1* and *HOIP* (135, 165). Patients with hypomorph LUBAC activity develop an immunodeficiency due to impaired NF-κB signaling. This happens especially in response to IL-1 and TNF. In addition, patients may develop amylopectinosis leading to muscular defects. Interestingly, these patients also develop multi-organ autoinflammation; perhaps this occurs because the patient’s monocytes exhibit an exacerbated pro-inflammatory response to IL-1.

Many animal models have been used to understand the physiological role of linear ubiquitin chains in the NF-κB pathway (**Table 2**). To fully appreciate the results of experiments with these animal models, it is important to understand that the expression of HOIL-1 is critical for the stability of HOIP and SHARPIN in murine cells. Additionally, the absence of either protein leads to greatly reduced expression of the other.

**Table 2:** List of mice models in linking NF-κB to autoimmunity.

Gene (protein)	Mutations	Phenotype	Refs
*TNAIP3* (A20)	-/-	lethal autoimmune disease	(167)
	B cell-specific deletion of A20	hyperesponsiveness of B cells and autoimmune disease with homologies to SLE	(168)
	dendritic cells-specific deletion of A20	enhanced activation of cDCs and moDCs. development of organ-specific autoimmunity but not systemic autoimmunity.	(169)
	myeloid cells-specific deletion of A20	intestinal pathology and cancer	(170)
	hematopoietic cells-specific deletion of A20	severe inflammation accompanied by B lymphocytes apoptosis	(171)
	epithelial intestinal cells-specific deletion of A20	dextran-sodium sulfate-induced colitis	(169)
	liver parenchymal cells-specific deletion of A20	chronic liver inflammation	(144)
	haplo-insufficient A20^-/-^ mice	psoriasis-like skin lesions associated with interleukin (IL)-17 and IL-23 overproduction	(172)
	A20-/- and A20-deficiency in astrocytes	neuroinflammation increase EAE disease severity	(173, 174)
*CYLD*	-/-	- T cell developmental defects and mice exhibit fewer mature CD4^+^ and CD8^+^ single positive thymocytes and peripheral T cells. - skin tumor development. - infection by *S. pneumoniae* infection - increase susceptibility to colitis-associated tumorigenesis following administration dextran sulfate sodium (DSS)	(175)
	B cells-specific deletion of CylD	no exacerbation of the developmental and activation defects of A20-deficient B cells	(129, 176–178)
	NKT cells-specific deletion of CylD	constitutive NF-κB activation responsible of a reduction of IL-7 secretion and ICOS expression	(178)
	skin-specific deletion of CylD	skin tumors after application of DMBA/TPA	(179)
*FAM105B* (OTULIN)	-/-	embryonic lethality	(180)
	inducible deletion of OTULIN in various lymphoid organs (spleen, thymus)	death of mice even in the adult state	(125)
	Bone marrow reconstitution experiments with inducibly depleted cells for OTULIN	- increase in neutrophils and cytokine secretion (TNF-IL-6, G-CSF) leading to systemic inflammation and autoimmunity - TNF neutralizing antibodies rescued the phenotype	(125)
	myeloid cells-specific deletion of OTULIN	- inflammatory phenotype - increased serum levels of inflammation-associated cytokines and chemokines. Mice develop splenomegaly and autoimmunity	(125)
*RBCK1* (HOIL-1)	-/-	polyglucosan body myopathy in old age	(154)
	-/- and TNF-/-, TNFR1-/-, RIPK3 -/- or Caspase 8-/-	- lethality mainly due to deregulation of TNFR1-mediated cell death. - Ripk3^-/-^Caspase 8^-/-^ HOIL-1^-/-^ embryos suffer from intrinsic defects in early hematopoiesis	(181)
*RNF31* (HOIP)	Transgenic expressing Deletion N-ter UBL HOIP	embryonic lethality at midgestational stage due to thoraco abdominal hemorrhages and vascular defects in embryo	(182)
	keratinocyte-specific deletion of HOIP and HOIL-1	severe dermatitis caused by TNFR1-induced, caspase-8-mediated apoptosis	(183)
*SHARPIN*	-/- SHARPIN -/- and TNF-/-	severe autoinflammatory disease (severe dermatitis and system-wide organ inflammation) and immunodeficiency rescued by TNF-/-	(23, 64, 184)

A *Hoil-1* ^-/-^ mouse model expressing a *Hoil-1* mutant deleted from its C-terminal ring domain exhibited polyglucosan body myopathy in old age (62). However, *Hoil-1* mutant mice with deletions in the N-terminal UBL exhibit embryonic lethality at the mid-gestational stage in mice (63, 67, 185). These mice presented thoraco-abdominal hemorrhages and vascular defects *in utero* (186). Additionally, they demonstrated that the UBL domain of HOIL-1 promotes HOIP function and is essential for linear ubiquitination in the TNF-SC (181). The lethality of these mice is mainly due to deregulation of TNFR1-mediated cell death since loss of TNF, TNFR1, RIPK3, or Caspase 8 reduced and delayed their lethality (181). Interestingly, *Ripk3* ^-/-^ *Caspase 8* ^-/-^ *Hoil-1* ^-/-^ embryos suffer from intrinsic defects in early hematopoiesis, resulting in deficiencies in erythroid and myeloid cells (181). The same happens on the skin where keratinocyte-specific deletion of *Hoip* and *Hoil-1* results in severe dermatitis caused by TNFR1-induced caspase-8-mediated apoptosis (183).

Furthermore, mutant mice lacking SHARPIN develop severe autoinflammatory diseases (severe dermatitis and system-wide organ inflammation) and immunodeficiency due to destabilization of the two remaining LUBAC subunits (23, 64, 184). These animals develop a phenotype known as chronic proliferative dermatitis. This condition is characterized by progressive epidermal hyperplasia, apoptosis of keratinocytes, cutaneous and systemic eosinophilic inflammation, liver inflammation, and loss of Peyer’s patches. As for *Hoip* and *Hoil-1*-deficiency, *Sharpin* deficiency-mediated dermatitis is remediated by *Tnf* ablation (187)

#### Deubiquitinases

##### CYLD

Many animal models have been developed to better understand the role of CYLD in the immune system. CYLD has been shown to promote the recruitment of the cytosolic kinase LCK to ZAP-70. It also plays a role in the positive regulation of TCR signaling (176). Consistently, KO mice for *Cyld* have T cell developmental defects and exhibit fewer mature CD4^+^ and CD8^+^ single positive thymocytes and peripheral T cells (188). Mice lacking *Cyld* are highly sensitive to skin tumor development (189). In B cells, the consequences of *Cyld* deficiency are controversial. It does not exacerbate the development and activation defects of *A20*-deficient B cells (177, 190, 191). Natural killer T (NKT) cells are a subset of innate immune cells that expresses semi‐invariant TCR-recognizing glycolipid antigens presented by the non‐polymorphic MHC class I‐related protein, CD1d (178). In these cells, *Cyld*-deficiency causes constitutive NF-κB activation which is responsible for a reduction of IL-7 secretion and ICOS expression, a costimulatory molecule that is typically expressed on activated T cells. ICOS is also crucial for NKT homeostasis and function (151). Consistent with its immune functions, loss of *Cyld* in mice causes *S. pneumoniae* infection (192) and increases susceptibility to colitis-associated tumorigenesis following administration of dextran sulphate sodium (DSS) (193).

The major function of CYLD is in the skin. Accordingly, transgenic mice overexpressing an inactive form of CYLD develop skin tumors after exposure to 7, 12-dimethylbenz(a)anthracene followed by 12-O-tetradecanoylphorbol-13 acetate (DMBA/TPA) (179).

##### OTULIN

Autosomal recessive hypomorphic loss-of-function mutations of *OTULIN* have been linked to an early-onset severe inflammatory disease, named OTULIN-related autoinflammatory syndrome (ORAS, also named Otulipenia) (125, 126). Patients with homozygous *OTULIN* mutations present with idiopathic systemic inflammation characterized by recurrent and prolonged fever episodes, joint swelling, diarrhea, dermatitis combined with panniculitis, neutrophilia, increased immunoglobulin levels in the blood, and autoantibodies (125). Patients’ leukocytes reveal increased NF-κB pathway activation and an accumulation of linearly ubiquitinated proteins. Levels of proinflammatory cytokines are considerably increased in stimulated patient’s mononuclear cells. While conventional anti-inflammatory therapy showed a poor response in ORAS patients, anti-TNF treatment reverses inflammation. Elimination of most signs of the disease was also observed after hematopoietic stem cell transplant (HSCT) in one patient, indicating that defective leukocytes are the main drivers in the development of ORAS disease. A recent study indicates that TNF-induced cell death in hematopoietic and non-hematopoietic cells may be at the origin of the disease (194).

Several mouse models of *Otulin* deficiency have shown that the loss of *Otulin* in mice is responsible for embryonic lethality (180). The inducible deletion of *Otulin* in various lymphoid organs (spleen, thymus) leads to the rapid death of mice, even in the adult state (125). However, conditional ablation of *Otulin* in immune cells, and, more specifically, in myeloid cells is sufficient to trigger several of the cardinal symptoms of ORAS.

##### A20

A20/TNFAIP3, is a remarkably potent regulator of ubiquitin-dependent signals; it has garnered much attention due to its link with human diseases (195, 196).

Truncating *TNFAIP3* mutations causes haploinsufficiency of A20 protein with upregulation of the NF-κB signaling pathway, NLRP3 inflammasome activation, and overproduction of proinflammatory cytokines. Patients present with the clinical feature of Behçet disease-like syndrome, a systemic vasculitis involving vessels of any size (197).

*TNFAIP3/A20* mutations have been reported in genome-wide association (GWAS) studies of SLE cohorts (198). Moreover, SLE has been described in one patient with a heterozygous loss-of-function mutation of the *TNFAIP3* gene (199). A *de novo* heterozygous frameshift mutation of *A20* was also associated in patients exhibiting the ALPS-like phenotype (200).

*A20*-deficient mice develop a lethal autoimmune disease, characterized by increased responsiveness to TNF-α- and TLR-mediated signals as well as spontaneous inflammation of multiple organs (154, 155). Several *A20*-conditional knockout mouse models have been generated. For instance, B cell-specific deletion of *A20* results in hyper-responsiveness of B cells and an autoimmune disease similar to SLE. The deletion of *A20* from dendritic or myeloid cells results in SLE-like or RA-like phenotypes (201, 202). Moreover, deletion of *A20* in hematopoietic cells exhibited severe inflammation accompanied by B-lymphocyte apoptosis (203). *A20*-deletion in epithelial intestinal cells results in DSS-induced colitis. Mice lacking *A20* in liver parenchymal cells develop chronic liver inflammation (204). More recently, it was found that haplo- insufficient *A20* ^-/-^ mice develop psoriasis-like skin lesions associated with IL-17 and IL-23 overproduction (172). The total or partial loss of *A20* results in neuroinflammation in mice, suggesting that A20 plays a key role in maintenance of nervous system homeostasis (205). *A20*-deficiency in astrocytes causes increased disease severity of experimental autoimmune encephalomyelitis (EAE; the animal model of MS) that correlated with increased NF-κB activity (168).

### Genetic Defect of Receptors Leading to Inflammation and NF-κB Deregulation

#### Intracellular Receptors

##### Inflammosopathies: NLRP3 and IL-1-Related Diseases

Activation of the NLRP3 inflammasome, a multiprotein complex, leads to caspase activation with production of proinflammatory IL-1β. This represents a major pathway of inflammation (206). Gain-of-function mutations of NLRP3 result in abnormal activation of the NLRP3 inflammasome and cause the autosomal dominant systemic autoinflammatory disease spectrum, termed cryopyrin-associated periodic syndromes (CAPS). Patients present with recurrent fever, urticaria-like rash, headache, conjunctivitis and arthralgia or arthritis. There are differences in the severity and length of disease flares, but all display serologic evidence of systemic inflammation (207). Among the CAPS syndrome, there are familial cold urticaria (FCU) and Muckle-Wells syndrome which are also associated with *NLRP3* mutations. This evidence leads to the conclusion that NLRP3 mutations can cause a variety of different phenotypes. Characterization of these mutations can provide new diagnostics tools and may suggest therapeutic target (208).

Pharmacological inhibition of NLRP3 activation results in potent therapeutic effects in a wide variety of rodent models of inflammatory diseases. These effects are mirrored by genetic ablation of *NLRP3* (209).

##### IL-1R Complex Related Diseases

Several autoimmune or inflammatory diseases are related to a dysfunction of the IL-1/IL-1R pathway. The recognition of this pathway involvement led to the use of anti-IL-1 antibody-based treatments.

- **Periodic fevers (not related to TNFR1 mutations)** can be accompanied with aphthous stomatitis, pharyngitis, and cervical adenitis (210–213). They involve increased IL-1β secretion even outside of seizures (214). Treatment is based on corticosteroids and IL-1β inhibitors (Anakinra).
- **Rheumatoid arthritis** is characterized by inflammation of the joints (215–217) In serum and synovial fluid, there is an increase in IL-1β (218). Extra-articular signs such as uveitis can be found. In this context, the increase in NF-κB activity is correlated with the symptomatology, which is improved by its inhibition.
**- Gougerot-Sjögren’s syndrome** is characterized by dry eyes and mouth. IL-1β levels are increased in serum and salivary secretions. It is thought to be responsible for inflammatory lesions and destruction of the salivary and lacrimal glands (219, 220). Anakinra could be a treatment for this disease (221).
- **Still’s disease** is characterized by febrile seizures and arthritis as well as rashes typical of inflammatory syndromes (222). The serum of these patients induces the production of IL-1β when incubated with peripheral blood mononuclear cells (223, 224).
**- Behçet’s disease** is characterized by bipolar ulcerations (mouth, genitals) as well as skin, eye, and joint lesions. There is an increase in IL-1β secretion in patients (225).
**- Type 2 diabetes** is characterized by chronic hyperglycemia. It increases the secretion of IL-1β and consequently the activation of NF-κB (226–228).

##### Interferonopathies

Type I interferon is involved in the induction and the end-result of a series of tightly regulated immune responses. Disturbance of this control, due to Mendelian mutations, causes type I interferonopathies (229). Some of these mutations occur on the gene *TMEM173* which encodes a stimulator of type I IFN gene (STING). Inherited *STING* activating mutations have been found in patients with lupus-like manifestations (230). IFNs activate not only the JAK/STAT signaling pathway, but also the NF-κB signaling pathway. NF-κB regulates the cellular response to IFNs (231). Heterozygous RelA mutations have been recently described in patients presenting early-onset systemic lupus erythematous.

#### Membrane Receptors

##### TNFRSF1A: Periodic Fever

TNF-Receptor-Associated Periodic Syndrome (TRAPS), which manifests as bouts of fever, and an aberrant inflammatory response leading to peritonitis, skin rashes, myalgias, and systemic amyloidosis has found new appreciation in the literature (232). TRAPS results from dominant mutations in the 55kDa TNF receptor gene (TNFRSF1A) disrupting the first two cysteine-rich region of the receptor. A mechanism that has been proposed of the inflammatory response in TRAPS centers around impaired TNFRSF1A cleavage, rendering the antagonistic soluble receptor unable to bind it (233).

## Basic Effector Mechanisms of Autoimmunity and Inflammation Related to NF-κB Pathway

### Relationship Between Neutrophil Extracellular Traps, NF-κB and Autoimmunity

Autoimmune diseases can arise from an imbalance between the formation and clearance of neutrophilis extracellular trap/NET (NETosis). Neutrophils, the most abundant white blood cells, have half-lives ranging only a few hours. They target and eliminate microbes by phagocytosis, degranulation, ROS (reactive oxygen species) release and the formation of NETs which are comprised of histones and chromatin. ROS, produced by mitochondria or by NADPH (nicotinamide adenine dinucleotide phosphate) are implicated in the progression of autoimmune diseases (234).

The prolonged formation of NETs is involved in autoimmune diseases due to the presence of pro-inflammatory proteins, such as IL-8, which recruit the neutrophils to the site of the NETs formation. Persistence of pro-inflammatory proteins in the NETs partially cause the organ lesions of many autoimmune diseases (235). NF-κB is implicated in generating DNA traps which can be reduced with NF-κB inhibitors (206).

### Autoimmunity, Autoinflammation, and Cancer

Uncontrolled NF-κB activation is a key link between autoimmune diseases and cancers. Observations of the presence of leukocyte in neoplasms were made by Virchow (236) and current literature estimates that around one in five cancers result from chronic inflammation caused by infections, toxins, and autoimmunity. In animal models, aberrant ROS and reactive nitrogen species (RNS) production have been shown to lead to colon cancer (237). The expression of inducible NO synthetase in macrophages and neutrophils triggers DNA damage through an NF-κB-dependent mechanism leading to tumorigenesis (238). By upregulating the secretion of pro-inflammatory cytokines, NF-κB plays a role in inflammatory bowel disease and recto-colic cancers (239). The role of NF-κB in tumorigenesis also varies based on cell type. While NF-κB activation in CD8^+^ lymphocytes and NK cells plays a role in tumor clearance in CD4^+^ T cells. This leads to neoplastic proliferation in CD4^+^ Foxp3^+^ regulatory cells for example. Inhibitory receptors like CTLA-4 and PD-1 which rely on NF-κB signaling for its expression can impair the immune defenses through specific exhaustion of CD8^+^ lymphocytes (118). Moreover, the production of cytokines such as TNF-α enables the proliferation of neoplasms, tissue invasion, and angiogenesis through the upregulation of IL-8, VEGF, and βFGF (240).

The role of TNF-α in tumor growth and its dependence on NF-κB signaling has been demonstrated in animal models (241). In humans, the inflammatory state of autoimmune illnesses such as SLE, rheumatoid arthritis, and IBD confer a significant risk for neoplasms (242). High levels of TNF-α, LPS, and IL-1 lead to a dysfunction of immune cells and an inability to control cancerous growth (243). In SLE, lymphocytes often have reduced activation capacity and the risk of tumorigenesis correlates with the stage of illness (242).

## Therapeutic Options Targeting the NF-κB Pathway

Most treatments proposed in autoimmune diseases inhibit the activation of immune cells and the signaling pathways of inflammation such as those activated by cytokines and their receptors. NF-κB has an important role in the pathophysiology of autoimmune diseases. Patients with autoimmune disorders may be cured with treatments that modulate the NF-κB pathway however, the overwhelming outcome of clinical trials on NF-κB selective compounds is for the moment non conclusive for autoimmune diseases (**Table 3**) (249). The most widely used drugs that modulate the activation of NF-κB are glucocorticoids, non-steroidal anti-inflammatory drugs (NSAIDs), and certain anti-rheumatic drugs. For example, Sulfasalazine acts on the nuclear translocation of NF-κB. Mesalamine inhibits post-transcriptional changes in p65 (102). High-dose aspirin inhibits the activity of IKKs (258). Additionally, glucocorticoids induce the transcription and synthesis of IκBα, thus increasing the cytosolic retention of NF-κB (259). Antibodies neutralizing cytokines or their receptors are also an effective treatment. For example, Anakinra (IL-1R antagonist), and Adalimumab (mAbs anti-TNF-α) can be used to treat autoimmune conditions. There are many ways to block the activation of NF-κB. Some are based on the use of broad-range therapeutics. Others are more specifically targeted. Among the broad-range inhibitors, those targeting the conserved adenosine triphosphate (ATP)-binding site of IKK molecules (SPC-839 and ML120B) have been shown to reduce the severity in preclinical models of rheumatoid arthritis (260, 261). Merck Sorono’s development of the SPC-839 however has been stopped. TPCA1, another NF-κB inhibitor, inhibits also STAT3 as well as decreasing the production of cytokines such as TNF-α, IL-6, and IL-8 (252). Another inhibitor, 3-s[(dodecylthiocarbonyl) methyl]-glutarimide (DTCM-glutarimide), exhibits anti-inflammatory activity *in vivo* and inhibitory effect towards receptor activator of nuclear factor-κB ligand (RANKL)-induced osteoclast differentiation in mouse bone marrow derived macrophages (253). While these inhibitors compete with the ATP binding site, another inhibitor, BMS-345541, has been described as a selective inhibitor of the allosteric sites of IKKα and IKKβ (it does not inhibit c-Jun and STAT3) (262). Prophylactic administration of this chemical product can prevent joint inflammation. It is also effective in murine DSS-induced colitis (263, 264).

**Table 3:** Therapeutic treatments targeting the NF-κB pathway.

Target in the NF-κB pathway	compounds	type	Mechanism of action	Ref.
**NF-κB**	Glucocorticoids	Anti-inflammatory	Binds to NF-κB and prevents transactivation. Induces transcription and synthesis of IκBα	(244, 245)
IKK	sulfasalazine	Anti-inflammatory	Inhibits IKKα and β activation	(246)
P65	Mesalamine	Anti-inflammatory	Inhibits IOL-1mediated p65 phosphorylation	(247)
IκBα	Aspirine (high dose)	Anti-inflammatory	Prevents IκBα degradation and suppresses NF-κB-dependent transcription	(248)
IL-1	Anakinra	Recombinant IL-1Rα	Prevents binding of IL-1β to its receptor	(249)
TNF-α	Adalimumab	Recombinant IgG1 mAb	Binds to TNF-α and prevent it to activate its receptor	(249)
IKKβ	SPC-839	Antiinflammatory	IKKβ inhibitor	(250)
IKKβ	ML120B	Antiinflammatory	IKKβ inhibitor	(251)
NF-κB	TPCA1	Antiinflammatory	Inhibits NF-κB and STAT3	(252)
RANKL	DTCM-glutarimide	Antiinflammatory	RANKL inhibitor	(253)
IKKα and IKKβ	BMS-345541	Antiinflammatory	IKKα and IKKβ inhibitor	(254)
NEMO	NEMO antagonist peptides	Peptides		(255)
Proteasome	Bortezomib	Cytotoxic agent (cell cycle arrest and apoptosis)	Proteasome inhibitor	(256)
Immunoproteasome	KZR-616		Immunoproteasome inhibitor	(257)
NF-κB	ODNi	Oligodeoxynucleotides	Inhibits binding of NF-κB to DNA	(255)

Another therapeutic strategy is to inhibit the interaction of IKK kinases with the regulatory element NEMO using cell-penetrating peptides interacting with the NEMO-binding domain of IKK (265). Encouraging results from this approach have been shown in a mouse model of DSS-induce colitis.

The ubiquitin-proteasome system plays an important role in enabling activation of NF-κB. The use of proteasome inhibitors like Bortezomib has been shown to be effective in murine models of LED, myasthenia gravis and colitis (266). A therapeutic trial (TAVAB) is currently underway on the effect of this treatment in refractory patients with LED, myasthenia gravis, and rheumatoid arthritis (170).

The immunoproteasome is expressed specifically in hematopoietic cells and is induced during the inflammatory process. Inhibitors that act specifically on subunits of the immunoproteasome have been developed and are a promising strategy to treat autoimmune diseases (267). KZR-616, a dual immunoproteasome β5i/β2i selective inhibitor developed by Kezar Life Sciences, has been recently approved for Phase II clinical trials for the treatment of several autoimmune diseases. Besides these approaches aimed at targeting kinase or proteasic complexes involved in the activation of NF-κB, another approach design to directly target the binding of NF-κB to DNA with synthetic DNA oligodeoxynucleotides (ODNs). This approach has not yet been used in patients, but its effectiveness has been shown in murine models of multiple colitis or arthritis (268). As described above, patients with common autoimmune disorders have a higher risk of developing hematological cancers or solid tumors. Autoimmunity is a factor of poor prognosis for these cancers. The risk of developing immune-related adverse events (IrAEs) is higher than in the general population (242). IrAEs appear due to therapy-associated cytokine release and T-cell infiltration when these cancers are treated with immunotherapy. Although the use of monoclonal antibodies targeting molecules that inhibit the immune system (CTLA4, PD-L1, PD-1) has been a revolution in the treatment of cancers, they cause IrAEs in 40% -60% of patients with cancer and autoimmune disease. For these reasons, patients with cancer are excluded from treatments with these checkpoint inhibitors because of the increased risk of toxicity. Nevertheless, patients with autoimmune diseases commonly use severe chemotherapy drugs as treatment for autoimmunity, such as cyclophosphamide, mercaptopurine, methotrexate, or mitoxantrone (269).

The NF-κB signaling pathway plays a key role in autoimmune disease through its action at several levels in immunological tolerance as well as its involvement in inflammatory lesions characterizing autoimmune diseases. Therefore, it is critical to develop treatments targeting specific effectors responsible for these diseases. Moreover, the fact that some immunotherapies are contraindicated in patients with autoimmune disease warrants the development of new agents to treat cancers that appear in patients suffering from these diseases.

## Conclusions

NF-κB plays a pleiotropic role in the immune system. It is active in different cellular compartments and tissues. In the thymus, it actively participates in central immune tolerance and is a key player in peripheral immune tolerance in secondary hematopoietic organs and circulating blood. NF-κB controls genes involved in anti-apoptotic mechanisms which play an important role in clonal selection. Furthermore, it is also involved in the migration of immune effector cells and in the inflammatory system by allowing the secretion of proinflammatory cytokines. It is not surprising that mutations affecting the function of effectors or loss-of-function mutations of negative regulators of the NF-κB pathway are responsible for autoimmune or inflammatory diseases.

In this review, we have described in detail the implication of different signaling pathways leading to NF-κB activation in immune tolerance and how the uncontrolled cytokine production resulting from genetic mutations of key effectors of these pathways can induce or aggravate autoimmune diseases.

## Author Contributions

All authors listed have made a substantial, direct, and intellectual contribution to the work, and approved it for publication.

## Funding

RW and EL are supported by the Centre National de la Recherche Scientifique (CNRS), Institut National de la Santé et de Recherche Médicale (INSERM) and Sorbonne University. LB is supported by the EUR G.E.N.E. program of the Université de Paris (reference #ANR-17-EURE-0013), by a government grant managed by the Agence National de la Recherche as part of the “Investment for the Future” program (Institut Hospitalo-Universitaire Imagine, grant ANR-10-IAHU-01, Recherche Hospitalo-Universitaire, grant ANR-18-RHUS-0010) and by a grant from the Fondation pour la Recherche Médicale (Equipe FRM EQU202103012670).

## Conflict of Interest

The authors declare that the research was conducted in the absence of any commercial or financial relationships that could be construed as a potential conflict of interest.

## Publisher’s Note

All claims expressed in this article are solely those of the authors and do not necessarily represent those of their affiliated organizations, or those of the publisher, the editors and the reviewers. Any product that may be evaluated in this article, or claim that may be made by its manufacturer, is not guaranteed or endorsed by the publisher.

## Glossary

AD: Autosomal Dominant
ANAs: Antinuclear Antibodies
ATP: Adenosine Triphosphate
BAFF: B Cell Activating Factor
BANK1: B cell scaffold protein with Ankyrin repeats
BCL2L: Human B cell CLL/Lymphoma 2 Like protein
BCR: B Cell Receptor
BS: Blau Syndrome
CAPS: Cryopyrin Associated Periodic Syndromes
CARD: Caspase Recruitment Domain
CBM: CARMA-BCL10-MALT1
CCDN1: Cyclin D1
CD: Crohn’s Disease
CPDM: Chronic Proliferative Dermatitis
CPG: Cytosine Phosphate Guanine
CVID: Variable Immunodeficiency
CXCL: Chemokine C-X-C motif Ligand
DAMPs: Damage Associated Molecular Pattern
DN: Double Negative
DP: Double Positive
DR: Death Receptor
DSS: Dextran Sulfate Sodium
EBV: Epstein-Barr Virus
EOS: Early-Onset Sarcoidosis
FCU: Familial Cold Urticaria
FOXP3: Forkhead box transcription factor
GOF: Gain of Function
GWAS: Genome Wide Association Studies
HVEM: Herpesvirus Entry Mediator
IKK: IκB Kinase
IL-1 β: Interlukine-1 β
IRAK3: IL-1 Receptor Associated Kinase 3
IRF4: Interferon Regulatory Factor 4
LOF: Loss of Function
LRR: Leucin-Rich Repeat
LTβ: Lymphotoxin β
MAPKs: Mitogen-Activated Protein Kinase
MDP: Muramyl Dipeptide
mTECs: medullary Thymic Epithelial Cells
NETosis: Neutrophil Extracellular Trap
NF-κB: Nuclear Factor-Kappa B
NFAT: Nuclear Factor Activated T cells
NIK: NF–κB–Inducing Kinase
NKT: Natural Killer T cell
NOA: NEMO Optineurin ABIN
NOD: Nucleotide-binding Oligomerization Domain-containing protein
ODNs: DNA Oligodeoxynucleotides
ORAS: OTULIN-Related Autoinflammatory Syndrome
OTU: Ovarian Tumor gene
PD1: Programmed cell Death-1
PHA: Phytohaemagglutinin
PIDs: Primary Immune Deficiencies
PMA: Phorbol Myristate Acetate
PRRs: Pattern Recognition Receptors
RIP4: Receptor-Interacting Protein kinase 4
RNS: Reactive Nitrogen Species
ROS: Reactive Oxygen Species
SHARPIN: SH3 and multiple ankyrin repeat domains protein (SHANK) associated RBPCK1 homology (RH)- domain interacting protein
SLE: Systemic Lupus Erythematosus
SP: Single Positive
STING: Stimulator of type I IFN Gene
TACI: Transmembrane Activator and Calcium modulator and cyclophilin ligand Interactor
TCR: T Cell Receptor
TIR: Toll-Interleukin 1 receptor
TLRs: Toll-Like Receptors
TNAIP3: Tumor Necrosis Factor Alpha Induced Gene 3
TNF: Tumor Necrosis Factor
TNFR: Tumor Necrosis Factor Receptor
TRAPS: TNF-Receptor Associated Periodic Syndrome
Tregs: Regulatory T cells
UBAN: Ubiquitin Binding in ABIN and NEMO
UBD: Ubiquitin Binding Domain
VEO-IBD: Very Early Onset-Inflammatory Bowel Disease
XHIGM: X-linked Hyper-IgM
